# Meanings within words: How Chinese bilingualism shapes the neural basis of English morphological processing in preschoolers

**DOI:** 10.1016/j.dcn.2026.101783

**Published:** 2026-07-09

**Authors:** Xin Sun, Janet F. Werker

**Affiliations:** a The Hong Kong Polytechnic University, Hong Kong; b University of British Columbia, Hong Kong

**Keywords:** Bilingualism, Preschool, Chinese-English bilingual, Brain development, Morphological processing, FNIRS

## Abstract

Preschool is a crucial developmental period during which children advance their spoken language skills that prepare them for later reading acquisition. During this period of rapid growth, bilingual experience fosters cross-linguistic interactions that shape distinctive neural architecture for language. This study explored cross-linguistic transfer by probing the developing bilingual brain basis for morphological processing, which emerges during the preschool years and supports reading development. While neural activation was measured with fNIRS, Chinese-English bilingual and English monolingual children (*N* = 155, ages 3–5) completed an English morphological word processing task that captures word roots (e.g., in compound words like ‘gold-fish’) and affixes (e.g., in inflectional and derivational words like ‘walk-ing’, ‘farm-er’). When processing word roots, bilinguals exhibited greater activation in the left middle temporal region, indicating enhanced semantic sensitivity compared to monolinguals. In contrast, when processing affix-based words, bilinguals showed greater left frontal activation, indicating enhanced morpho-analytical processing. Moreover, the brain-behaviour associations aligned with bilingual children’s experience with Chinese, a language characterized by semantically transparent compound structures and the relatively limited derivational morphology. Higher Chinese proficiency was associated with *decreased* left inferior frontal activation for English word roots, suggesting that Chinese experience facilitates access to word roots. In contrast, higher Chinese proficiency was associated with *increased* left inferior frontal activation for English affixes, suggesting that Chinese experience yields enhanced analytical demands for affixes. These results strengthen evidence of cross-linguistic transfer in the preschool years, and highlight how spoken language experience sculpts the brain for later language and literacy development.

Early childhood is a crucial period for language and literacy development. During this time of rapid development, a child’s brain undergoes significant changes, and linguistic experiences actively sculpt the neural architecture for language processing (Werker and Hensch, 2015). In particular, for children with dual language experience, theories of bilingual transfer suggest that their two languages interact to yield unique neural pathways for some aspects of language processing (Cargnelutti et al., 2019, Chung et al., 2019, Liu and Cao, 2016, Sulpizio et al., 2020, Zhang et al., 2023). Emerging differences in neural organization have ramifications for both language learning and language comprehension. To advance the understanding of how bilingual experience sculpts the brain, the current study focuses on the influences of Chinese-English bilingualism on the neural basis for morphological word processing in preschool-age children, who are at the critical interface of spoken language acquisition and formal reading instruction.

## 1 Background and motivation

### 1.1 Cross-linguistic interactions on bilingual morphological development

Words consist of one or more meaning units, known as morphemes. Common word morphology rules include compounding, inflection and derivation. For example, ‘snowman’ is a compound word consisting of two root morphemes; ‘cats’ is made by attaching the inflectional affix morpheme ‘-s’ to the root ‘cat’; and ‘singer’ is a derivation from ‘sing’, adding the derivational affix ‘-er’. While both English and Chinese employ these morphological rules to create words, their vocabularies favour different types of morphological structures (McBride-Chang et al., 2008). Chinese words are primarily made with compounding; indeed, over 70% of modern Chinese words are lexical compounds (Tse et al., 2017, Wei et al., 2023), whereas in English, derivations and inflections are more common. For example, the English word ‘washer’ is made up from the lexical root ‘wash’ and the derivational affix ‘-er’. In Chinese, this same “word” is comprised of three lexical roots, 洗衣机 (wash-clothe-machine, xi3 yi1 ji1). Other examples include the word ‘dishwasher’ 洗碗机 (wash-bowl-machine, xi3 wan3 ji1) and the word ‘dryer’ 烘干机 (heat-dry-machine, hong1 gan1 ji1). These language-specific morphological structures raise the important question: for a child growing up in both Chinese and English environments, how does exposure to these contrasting morphological distributions influence the neurocognitive profiles for language development?

Theories of bilingual acquisition posit that children transfer skills learned from one language to the other (Interactive Transfer Framework, Chung et al., 2019). Such transfer effects typically happen at the shared linguistic aspects between bilinguals’ two languages. Transfer of a linguistic aspect often occurs from the language featuring such a form to the language in which that form is less common. In terms of morphological structures, Chinese and English both have compounds, and studies have shown robust concurrent and longitudinal associations between school-age children’s Chinese and English compound awareness (Ke et al., 2021, Luo et al., 2014). However, as noted above, compounding is a stronger feature of Chinese compared to English. Consistent with a language transfer prediction, Chinese compound awareness has been found to statistically predict English reading proficiency, above and beyond the effects of English vocabulary and morphological awareness, yet this statistical prediction does not run in the opposite direction (Zhang and Koda, 2014). Moreover, proportionately more English words are created from affixes, and Chinese has relatively limited derivational morphology. These cross-language morphological features influence children’s proficiency with English compounds and derivations. For example, in a study with 4th- and 7th-graders, Ramierz and colleagues (2011) found that Chinese-English bilinguals showed equivalent proficiency to monolinguals in English compound awareness tasks; however, both 4th- and 7th-graders bilinguals scored significantly lower than English monolinguals in the derivational awareness tasks. Many other studies have also revealed that Chinese-speaking children find English affixes more challenging (Ku and Anderson, 2003, Paradis et al., 2016).

### 1.2 Preschool as an essential stage of English morphological development

While children begin to show sensitivity to morphemes from infancy, it is not until preschool years that children start to flexibly use different types of morphemes to understand and produce new words (Fejzo et al., 2018, Kim and Sundara, 2021, Shi, 2014, Waxman et al., 2009). In one widely cited study (Clark et al., 1985), less than half of the two-and-a-half-year-old toddlers understood ‘mouse-hat’ means ‘a hat on a mouse’, yet 84% of preschool children passed this task. By the preschool years, children can also fluently use simple and consistent inflectional morphological structures, and can -- for example, attach ‘-ing’ to verbs and plural ‘-s’ to nouns, and demonstrate competence in manipulating simple derivational morphemes like ‘-er’ (Berko, 1958, Clark, 2017, Nicoladis, 2006, Wagner et al., 2009; see also Clark, 1993, Fejzo et al., 2018). For example, Lam and Sheng (2016) found that 4-to−5-year-old English preschoolers achieved high accuracy (∼80%) in a morphological analogy task, which asked children to produce compounds like ‘snowman’ and ‘-er’ derived words like ‘writer’.

Preschoolers not only begin to show skills in manipulating morphemic structures, but their morphological skills at this stage also predict language outcomes. A recent study has shown that both compound and affix awareness are significantly associated with preschoolers’ vocabulary sizes (Sun et al., 2025). This pattern differs from what is typically found with school-aged children, where derivational awareness plays a more prominent role in language and literacy. By the time children enter school, inflectional affixes and compounds evident in the preschool years become more automatic, while derivational forms become more challenging due to increased exposure to a richer and more complex set of derivational affixes through formal reading instruction (e.g., “-tion” and “dis-”, Anglin et al., 1993; Korochkina et al., 2025; Kuo and Anderson, 2006). Consequently, in school-aged children, significant associations are often found between derivational morphology mastery and reading achievement, with weaker links between reading and compound awareness (Korochkina et al., 2025, Levesque and Deacon, 2022, Liu et al., 2024, Marks et al., 2022). These findings together suggest that formal reading instruction introduces a developmental shift in morphological skills, with preschool oral language serving as a foundational stage in which specific morphological structures uniquely contribute to a child’s language competency.

### 1.3 How the brain represents multi-morphemic words

Neural processing of word morphology involves an integrative representation of phonological, semantic, and syntactical analysis, and thus engages a distributed but interconnected set of language networks (Cavalli et al., 2016). Informed by adult studies, Gwilliam (2020) proposed a multi-stage framework of morphological comprehension: morpheme segmentation, lexical access, and morphological composition. Morpheme segmentation refers to recognizing the morphemic units within a word. It is essentially a phonological segmentation process and accordingly primarily involves the auditory cortex, or the Superior Temporal Gyrus (STG, Cavalli et al., 2016; Ettinger et al., 2014; Gwilliams and Marantz, 2015). Lexical access connects the segmented morphemes to their corresponding semantic and syntactic features. Here, different morphemes may require specific brain involvement. Root morphemes are the most lexically transparent units (e.g., ‘snow’ and ‘man’ in ‘snowman’) and engage the semantic regions located in the Middle Temporal Gyrus (MTG, Friederici, 2012; Hickok and Poeppel, 2015). For example, Zou et al. (2016) tested adult participants with a word similarity judgment task and found that word pairs with shared root morphemes activated stronger left MTG and IFG compared to pairs of repeated words (see also Hsu et al., 2019). Affixes (i.e., inflections and derivations) provide additional semantic and grammatical characteristics about the word. In particular, inflections specify grammatical information like quantity (e.g., plural ‘-s’), and derivations modify the word by changing part of speech and adding non-grammatical meaning (e.g., ‘-er’ in runner means someone who runs). Accessing affixes involves morpho-syntactic processing and engages the Inferior Frontal Gyrus (IFG, Bulut, 2022; Gao et al., 2023; Leminen et al., 2019) as well as the Middle Frontal Gyrus (MFG, Bick et al., 2010; Shapiro and Caramazza, 2003). Derivational affixes, compared to inflectional affixes, demand stronger analytical brain resources as recorded in the frontal lobe. In illustration, when asked to produce root word from a given derivation (e.g., given ‘failure’, produce ‘fail’), participants engaged more left IFG, MFG, and parietal regions than when an inflectional word cue was given (e.g., given ‘played’, produce ‘play’, Marangolo et al., 2006). In all, dealing with morphologically complex words is often a multi-stage process and engages interrelated yet different neural mechanisms.

Developmental research has focused not only on comparing child neural organization for morphological processing to that of adults, but also on how neural activation to simple and complex morphology is related to language and reading development. Similar to the behavioural literature, most of this work has focused on school-age samples and mainly explored brain-behaviour relations related to the morphological structures key to reading in children’s dominant language. Arredondo et al. (2015) tested 6–12-year-old English-speaking children with a derivational word judgment task (i.e., ‘rejump’ is a valid derivation for ‘jump’, yet ‘re-apple’ is not valid for ‘apple’) and found significant left STG, IFG, MFG and bilateral Superior Frontal Gyrus (SFG) activation compared to control (i.e., word repetition). The MFG and SFG activation revealed the higher-level cognitive mechanisms involved when children access English derivations. Further, individual differences in English literacy are associated with differential brain activation during derivational tasks. For example, Marks et al. (2022) found that stronger reading comprehension skills were associated with stronger activation in the left IFG, MTG, and parietal regions during a derivational word matching task. In contrast to the focus on derivational forms with English children, research with Chinese children has also looked at the processing of the dominant Chinese morphological form, compounds. Prior research has shown that the neural circuits for compounds distinguish children with dyslexia from typical controls (Liu et al., 2013). Compared with typical controls, children with dyslexia were less sensitive to morphologically related words (i.e., ‘wine’ 葡萄酒 and ‘liquor’ 白酒, sharing a root morpheme 酒 in Chinese) compared to words with only semantic relations (i.e., ‘forest’ 森林 ‘beast’ 野兽), with corresponding smaller activation differences in the left IFG.

### 1.4 Morphological processing in the bilingual developing brain

Theories of bilingual neural development suggest that variations in language properties, proficiency, and exposure interact with the maturation of the brain and jointly shape the bilingual neural circuits underlying language and future literacy (Hernandez et al., 2019; Kovelman & Sun, in press; Yeh et al., 2026). The Neuroemergentism Framework offers a broad stance that the bilingual brain is shaped by children’s dynamic interactions with the input languages throughout development. More specifically, in their recent review, Yeh et al. (2026) point out that developmental dynamics (i.e., age of acquisition) play an essential role in shaping the bilingual brain: while second-language learners rely on neural adaptation mechanisms in response to language input, early-immersed bilinguals show on-going neural refinement leading to more efficient networks. Moreover, in their Bilingual Reading Brain Framework, Kovelman and Sun (in press) document how bilingual experience influences the brain by highlighting person-specific factors including proficiency and age of acquisition, and importantly, the language-specific factors. These language-specific factors tap into cross-language similarities and differences in key aspects of language and reading acquisition, such as morphological structures and decoding principles. In sum, early bilingualism shapes the brain and language properties, such as morphology, which contribute to this dynamic, interactive process of bilingual neural development.

Empirically, a few neuroimaging studies to date have investigated the effect of bilingualism on morphological processing in school-age children (Ip et al., 2017, Sun et al., 2022, Sun et al., 2023). These studies echo findings from the behavioural literature and show interactions between children’s morphological competency and bilingual exposure. For example, Sun et al. (2022) found that, in both monolingual and bilingual children, accessing derivational affixes elicited stronger left IFG and STG activation than accessing word roots. This aligns with school-age children’s morphological competency, as compounds become more automatic than derivational affixes after entering school. Interestingly, bilingualism was also found to interact with morpheme type: compound words were equally automatic to bilinguals and monolinguals, with no statistical differences in brain activation; however, derivational affixes activated stronger left IFG and MFG in bilinguals than monolinguals. The additional frontal region involvement revealed cross-linguistic impacts, indicating that experience with Chinese, a language with relatively limited derivational morphology, is associated with higher demands on analytical brain resources for language processing. In a final finding, bilinguals’ Chinese literacy was associated with their STG activation only during the derivational but not the compound words (Sun et al., 2022). Together, these findings show that school-age bilinguals, who already have formal reading instruction in English, approach English lexical morphology with influences from exposure to morphological forms in both of their languages.

The cross-linguistic interactions in school-age children’s reading brains pose a question about the preschoolers' listening brains. In other words, what is unknown is the neural organization of morphological processing at the critical juncture when preschoolers have begun to use different types of morphemic units, but have not yet had the formal reading instruction that makes morphological skills more explicit. Here, we address the gap and ask whether experience with the compound-heavy Chinese language yields stronger automaticity in processing English compounds already in the preschool years. Moreover, as noted above, while English preschool-aged children are beginning to use the relatively straightforward affix structures (including inflections and derivations) at this time, Chinese lacks affix structures. Thus, we ask whether the use of affix structures interacts with Chinese-English bilingualism to yield distinct patterns of brain activation in comparison to that seen in English monolinguals. Finally, we further examine these cross-linguistic interactions by looking at whether individual differences in bilingual proficiency are associated with individual differences in the neural bases of morphological processing.

We address these questions with a direct comparison of spoken English morphological processing between Chinese-English bilingual and English monolingual preschoolers using functional Near-Infrared Spectroscopy (fNIRS) neuroimaging. Together, our inquiry into preschoolers, who are positioned right before the onset of formal reading instruction, offers an opportunity to understand the language-specific effects of mono- vs bilingual spoken language development on the neurobiology of morphological processing at a key period of morphological acquisition.

### 1.5 The present study

The present study examines the brain basis for morphological processing of spoken language in Chinese-English bilingual and English monolingual preschoolers. In particular, working from a language transfer perspective, we are interested in how bilingual experience with Chinese, a language with extensive compounding structures and relatively limited derivations, influences preschoolers’ neural architecture for English morphological root/compound and affix processing. To address this question, we tested 3-to−5-year-old preschool children who had sufficient exposure to either both Chinese and English or only English, with a word morphology task during fNIRS neuroimaging. To measure the brain functions for specific types of morphemic structures, the word morphology task included two experimental conditions to probe root/compound and affix processing, respectively. To understand how bilingual proficiency is associated with the brain basis for morphological processing, participants also completed behavioural language assessments. The behavioural measures also allowed us to determine whether the two groups possess similar English proficiency.

Several analyses were conducted. First, in Model Set 1, we mapped the brain regions associated with root/compound and affix processing for each language group, and conducted a direct contrast of these brain bases between Chinese-English bilinguals and English monolinguals. We hypothesized that Chinese-English bilinguals would show more automated MTG neural activation during the root/compound condition than English monolinguals. In contrast, English monolinguals were predicted to show more automated neural activation during the affix condition than the Chinese-English bilinguals, specifically in the inferior and middle frontal regions. In Model Set 2, we examined how the brain activation for morphology may correspond to individual differences in children’s English morphological proficiency, as probed in a brain-behaviour correlation analysis. In Model Set 3, we examined the potential bilingual interactions through cross-language brain-behaviour associations between bilinguals’ brain activation during the English morphology task and their Chinese language proficiency.

## 2 Method

### 2.1 Participants

The final sample included *N* = 155 children (68 girls) aged 3 years 9 months to 5 years 11 months, *M(SD)*_*Age*_ = 60.6(7.11) months from the [blinded for review]. All participants were raised in Canada and attended English-only preschools. Before participating in the study, parents were screened with a question to estimate their child’s overall language exposure: “What percent of the time does your child hear English?”. During the study, parents also completed a comprehensive interview to report their child’s language exposure from birth to the present (Adapted from Byers-Heinlein et al., 2020). Both the one-question global estimate and the cumulative exposure estimate from the interview were used to determine children’s bilingual status. Based on these responses, participants were categorized as monolingual if parents reported 90% or more exposure to English in one of the above measures. There were *n* = 71 English monolingual children, *M(SD)*_*Age*_ = 61.06(7.15) months, 32 girls. According to the parent global estimate, the overall English exposure was *M(SD)* = 96.75% (4.41) of the time for monolinguals. Their cumulative English exposure was *M(SD)* = 95.14% (9.32) of the time. Chinese-English bilingual participants were heritage speakers born to and raised by at least one parent who speaks Chinese as their native and most proficient language, and reported having exposure to Chinese at least 20% of the time on at least one of the measures above. There were *n* = 84 Chinese-English bilinguals (*M(SD)*_*Age*_ = 60.21(7.1) months, 36 girls). Chinese-English bilingual children were exposed to Mandarin (*n* = 51) or Cantonese (*n* = 33). According to the parent global estimate, the overall English exposure was *M(SD)* = 51.04% (20.68) of the time for bilinguals. Their cumulative English exposure was *M(SD)* = 40.35% (19.73) of the time for bilinguals. Monolinguals and bilinguals were matched on age (*t*(153) = -.71, *p* = .465). The study was approved by the university Ethics Review Committee (H22–02731).

### 2.2 Measures and procedure

All participating children completed a battery of standardized or experimenter-developed language assessments in English. The standardized tests assessed phonological awareness (blending subtest of the Comprehensive Test of Phonological Processing, CTOPP; Wagner et al., 1999), receptive vocabulary (Peabody Picture Vocabulary Test 5, PPVT−5; Dunn, 2019), and letter-word reading (Letterword Identification subtest, Woodcock-Johnson IV, WJ-IV; Schrank et al., 2014). Experimenter-adapted unstandardized assessments included tests of morphological awareness. The Chinese-speaking participants also completed several tasks in Chinese, including Chinese morphological construction and vocabulary. The unstandardized assessments are introduced below.

*English Morphological Awareness* was assessed using a subset of the morphological production task by Lam and Sheng (2016) designed for 4- to 8-year-olds. For each item, a child was first shown a picture and asked to name the picture with morphological regularities. An example item was a picture of a bear painted brown. The experimenter would point to the picture and say, “This is a bear with brown fur. What do we call a bear with brown fur? We call it a ___. (brown-bear).” The internal consistency of this task is greater than.90 according to Lam and Sheng (2016).

*Chinese Morphological Awareness* used a morphological production test adapted from Sun et al., 2021. The items were selected based on our pilot data to match the word knowledge of this age group. Children are instructed to create a new word with the morphemes in a given word. Pictures are shown to help children understand each item. For example, “如果有张纸是白色的，我们就会叫它白纸，如果有张纸它是红色的，我们叫它？（红纸）” (If this piece of paper that is white is called white paper, then if there is a piece of paper that is red, then we call it …? Red paper). The internal consistency of the current sample is.95.

*Chinese Vocabulary* was assessed using a translated version of PPVT−5. The measure had a Mandarin and a Cantonese version. The translation was completed by the first author, bilingual speaker of Mandarin (native) and English (second language), a bilingual native speaker of both English and Cantonese, and a trilingual speaker of Cantonese (native), Mandarin (native), and English (second language). Participants start from words of their age and stop when they make 6 consecutive errors. For each item, children were shown four pictures and asked to pick the one that best described a word. The measure showed strong validity with a bivariate correlation of *r* = .71 (*p* < .001) and age-controlled partial correlation of *r* = .68 (*p* < .001) with the Chinese morphological awareness measure. The internal consistency of the current sample is.99.

### 2.3 fNIRS neuroimaging morphological processing task

The neuroimaging morphological processing task includes two morphological experimental conditions and one control condition. The experimental conditions tap into free root morphemes (as in root-based and compound words) and affix morphemes (as in affix-inflection and derivation-based words), respectively. The control condition is a whole-word match task that does not require morpheme segmentation and semantic retrieval. Across all three conditions, each trial included three auditorily presented words, with one target word and two words of choice.

#### 2.3.1 Free roots/compounds experimental condition

In the free roots/compound trials, the correct answer shares a morphemic root with the target/first word. For example, when presented with target *bee,* plus *bumblebee,* and *frisbee*; the correct answer is *bumblebee*, which shares a root morpheme with the word (also a root morpheme) *bee.* The phonological distractor *frisbee is* an incorrect choice that only shares phonological similarity with the first/target word.

#### 2.3.2 Affixes experimental condition

In the affix trials, the correct answer shares either the derivational affix ‘-er’ or the inflectional affix ‘-ing’ with the target/first word. For example, *swimming*, *walking,* and *something*. The correct answer is *walking*, with the -*ing* denoting a verbal action, as in the target word *swimming*. On the other hand, the phonological distractor *something* is an incorrect choice. An example with the ‘-er’ affix is *singer, reader, corner*.

#### 2.3.3 Control word recognition condition

The control condition was a passive word processing task in which children were expected to select the complete match to the target rather than shared morphemes (e.g., *welcome*, *welcome*, *cartoon*). The condition ensured that participants were on-task during the session.

### 2.4 Morphological processing task stimuli

Across three conditions, words were matched in the number of letters, phonemes, syllables, and morphemes. Across all, words on average have *M(SD)* = 6.21(1.57) letters, *M(SD)* = 5.19(1.45) phonemes, *M(SD)* = 1.87(0.55) syllables, and *M(SD)* = 1.45(0.52) morphemes. Words were also matched in the age of acquisition, which was determined by the Auditory English Lexicon Project database (AELP, https://inetapps.nus.edu.sg/aelp/; Goh et al., 2020) and Kuperman et al. (2012). The age of acquisition across all words is *M(SD)* = 4.69(0.92) according to ALEP, and *M(SD)* = 4.78(0.89) according to Kuperman et al. (2012). Specific data on the number of words, phonemes, syllables, morphemes, and age of acquisition are shown in Supplement Table S1. One-way ANOVAs between words of the three conditions revealed no significant differences in any of the parameters above (all *p*s > 0.05).

### 2.5 fNIRS neuroimaging task procedure

**Fig. 1:**
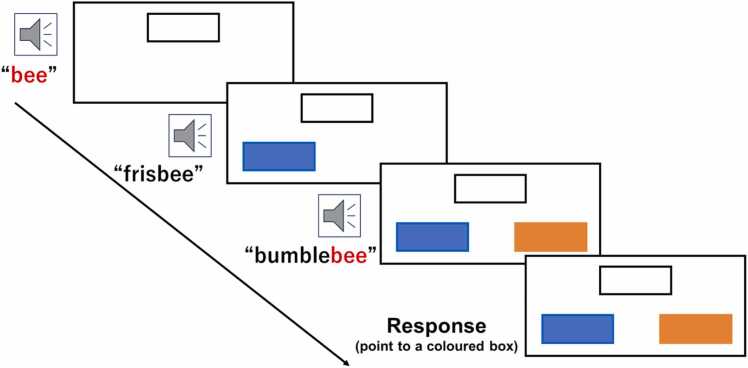
A sample trial of the fNIRS morphological processing task. *Note.* For each trial, participants first hear the target word (e.g., “bee”) and are shown a white box in the middle top of the screen, then participants hear two words (e.g., “frisbee,” “bumblebee”) and see a blue and an orange box as they hear the audio, respectively. Because the task was purely auditory, coloured boxes were used as visual aids on the screen to help children gauge each word and indicate their responses by pointing to the coloured boxes.

The neuroimaging task was programmed by PsychoPy and presented on an iPad. Participants were instructed to use the Apple Pencil to point to and select their choice. Before the task, participants underwent a training session to familiarize themselves with the iPad interface and the task. The instructor introduced the task as a “word-matching game” and guided participants through five example trials. In each trial, participants heard three words and were shown three pictures. The first word spoken was the target word, and the picture was shown in the top middle of the screen (e.g., “bedroom”). The second and third words were the two words of choice, and the pictures were on the bottom left and bottom right of the screen, respectively (e.g., “classroom”, “mushroom”). Participants were then asked to point to the picture/word that matches the first picture (i.e., the target). The picture practice trials included all possible types of word conditions, i.e., free roots/compounds, the affix ‘-ing’, the affix ‘-er’, and the control whole-word match. The experimenter ensured that the participants understood the task and the answers by discussing the correct answer with the participant if they had picked the incorrect picture. Next, children were instructed to complete more practice trials without pictures, but with only rectangles to correspond to the words they had heard (i.e., identical to the experimental trials as shown in Fig. 1). After each practice trial, children were corrected with explanations if they pointed to the incorrect box. Children would only proceed to the main task upon full understanding of the task procedure.

The main task consisted of 12 blocks and a total of 48 trials. Each of the three conditions had four blocks, and each block had four trials, making each condition 16 trials in total. The number of blocks and trials were designed based on prior fNIRS literature with child populations (Sun et al., 2023, Xie et al., 2021). Each block lasted 30 s, and there was a 9-second rest in between blocks. The whole task lasted a total of ∼7.7 min. Task trials were presented as follows (also see Fig. 1). Three spoken words were presented sequentially, first the target word, and then the two words of choice. Upon the oral presentation of each word, a rectangle appeared on the screen. The first/target word was paired with a blank rectangle in the top middle of the screen. The second and third words were paired with a yellow and a blue rectangle on the bottom left and bottom right of the screen, respectively. Participants were asked to use the Apple pencil to point to either the yellow or the blue rectangle to indicate their word choice. The block order and item order within blocks were randomized once (see Supplement Table S1 for the item and block sequence).

Participants’ responses were recorded manually by the experimenter who stood behind the child during the fNIRS session. This approach was used to accommodate the developmental constraints of this young age group. During the pilot stage, we found that children at this age often struggled with the traditional keyboard or button box, as they had difficulties in mapping the keys to the words on screen. To make the task age-appropriate, we asked children to indicate their choice by pointing to their answer (i.e., the rectangle) on the screen. While screen touches can be recorded from the Psychopy program via Apple pencil, many children indicated their choices by pointing close to the screen without making physical contact with it. This made the program unable to reliably capture children’s responses. We therefore recorded participants’ answers via a scoring sheet by an experimenter. Because of this, precise reaction time metrics could not be captured and we only reported task accuracies.

### 2.6 fNIRS setup and data acquisition

**Fig. 2:**
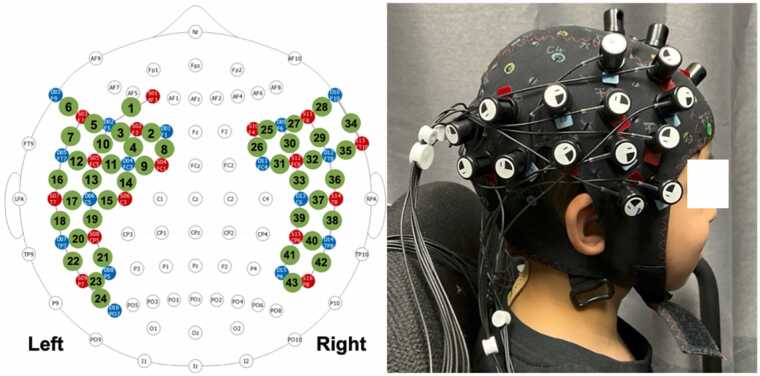
fNIRS cap configuration (left) and demo participant picture (right).

**Fig. 3:**
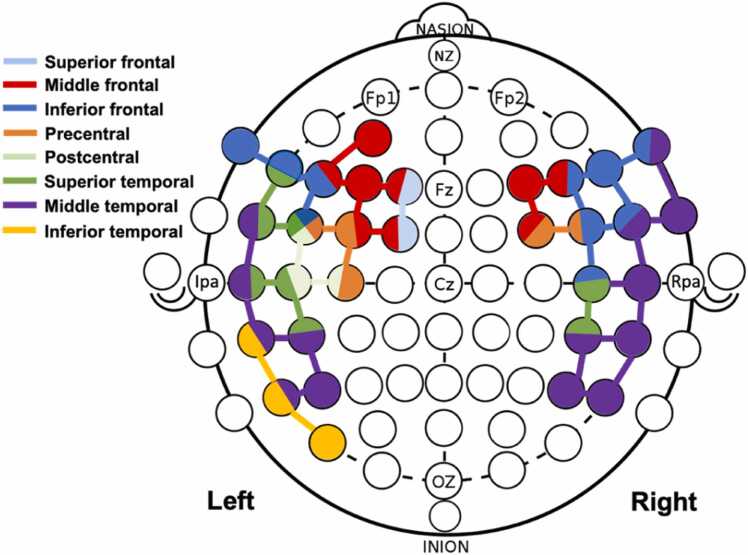
fNIRS channel localization. *Note.* Each circle indicates a light sensor (source or detector) that corresponds to the red and blue circles in Fig. 2. Each line connecting two circles indicates a channel that corresponds to the numbered green circle in Fig. 2.

fNIRS data were collected with the NIRSport2 system from NIRX (www.nirx.net). The NIRSport2 device has source wavelengths of 760 nm and 850 nm and a sampling frequency of 5.1 Hz. The cap configuration used all 16 sources and 16 detectors from the fNIRS device, with 9 sources and 9 detectors on the left hemisphere, and 7 sources and 7 detectors on the right hemisphere (Fig. 2). These light sensors together yielded 43 data channels, 23 on the left hemisphere and 20 on the right hemisphere. We used fNIRS caps that varied in size from 48 cm to 54 cm to accommodate the different head sizes of the children tested. The caps were made of cloth with moderate stretch and were provided by NIRX from EasyCap (www.easycap.de). We designed the fNIRS cap configuration to cover key language processing areas, including bilateral frontal and temporal regions. The light sensor locations were placed based on a channel placement template from the devFold Matlab toolbox (Fu and Richards, 2021). devFold uses MRI scans and co-registered locations of all possible source-detector channel combinations placed on a standard 10–10 EEG cap. It utilized MRI scans from different age groups and provided fNIRS channel placement templates from infancy to adulthood. The current study built the fNIRS cap configuration based on the 4-year-old template. The probe configuration was initially built to target key regions of language processing, including bilateral IFG, MFG, STG, and MTG with a 30% specificity in devFold. This specification yielded a layout of 21 sources and 19 detectors. To accommodate the 16 × 16 optode limit of our fNIRS system, we removed 5 sources and 3 detectors that had the lowest specificity of target ROIs. The MNI coordinates and brain regions of each channel were retrieved from the devFold toolbox. The regions of each channel are depicted in Fig. 3, and the MNI coordinates are attached in Supplementary Table S2. We acknowledge that, due to the nature of the fNIRS technique, here and henceforth, our results and discussions of the brain regions refer to maximal anatomical overlays of the channels of our cap configuration (Hu et al., 2020).

Trained experimenters followed standard written scripts (adapted from www.easycap.de) to conduct head measurement and place the fNIRS cap onto each participant’s head to ensure consistency. The experimenters first located participants’ nasion, inion, left and right preauricular points, Fpz, Fp1, Fp2, and Cz, then measured left-to-right preauricular distance and head circumference. Next, experimenters selected the cap based on the head circumference (i.e., 48 cm, 50 cm, 52 cm, and 54 cm). Finally, experimenters carefully applied the cap onto the participant’s head based on the marked anchors described above.

Once the cap was in place, the research team adjusted the setup for optimal signal quality via the following steps. The fNIRS system (operated with the Aurora software from NIRX) was launched, and signal optimization was conducted. If necessary, experimenters tried to attach the sensor closer to the head scalp by clearing hair around the sensors and/or using stronger spring tops to push the sensor closer to the skin. The team repeated signal optimization until achieving an acceptable signal-to-noise ratio.

### 2.7 fNIRS data processing and statistical analysis

Before proceeding to fNIRS data analysis, an inclusion criterion regarding fNIRS task accuracy was applied to ensure participant engagement while also considering participants’ young developmental stage. Specifically, a minimum accuracy of 50% in at least one of the experimental conditions was required as an indicator of active engagement with the experimental stimuli. This criterion was based on prior work, although we adopted a more lenient threshold given participants’ age. Based on this criterion, eighteen bilinguals and sixteen monolinguals were excluded from the analysis.

fNIRS data analyses were conducted using the NIRS Brain AnalyzIR, a MATLAB-based toolbox from Santosa et al. (2018), and experimenter-developed scripts (adapted from https://github.com/xiaosuhu). Before formal analysis, several data-quality checks were conducted to remove low-quality data. Specifically, we checked for valid heart rate, motion artifacts, and adequate signal-to-noise ratio (based on https://github.com/xiaosuhu/fNIRS-Dataqualitycontrol-for-Nirstoolbox). Three bilinguals and four monolinguals failed the data-quality check and thus are removed from the subsequent analyses.

#### 2.7.1 Subject-level analysis

For each participant, the raw fNIRS data were first trimmed to keep 5 s of pre- and post-task data as a baseline. Next, optical density data were converted to hemoglobin concentration by applying the modified Beer-Lambert law. Hemoglobin concentration data were then modelled with the general linear model (GLM; Friston et al., 2007). Motion corrections were conducted with an autoregressive-whitened robust regression solution as indicated in Barker et al. (2013). The canonical hemodynamic response peaking 6-s after trial onset was used as the basis function for the modelling process (Friston et al., 2007). With this process, results yielded regression coefficients (beta values for each task condition) for HbO (oxygenated hemoglobin) and HbR (deoxygenated hemoglobin) signals of each channel and each participant. The distribution of beta values of the significant channels for each language group and task condition is attached in the Supplementary Materials Figure S1.

#### 2.7.2 Group-level analysis

For each channel, group-level analyses were conducted using the linear mixed-effects (LME) models. The first set of models aimed to map out the brain activation for different morphological structures by language group. We fitted a model using task condition (Free roots/compounds, Affixes, and Control), language group (English monolingual and Chinese-English bilingual), and their interaction to predict the individual-level HbO and HbR beta values from the subject-level analysis. The LME formula for this model is “beta ∼ condition*language group + (1|Participant).” The brain basis for morphological processing for each participant group was planned to be calculated through the group-level contrasts between experimental and control conditions (Task vs Control) for each channel. Unique brain involvement in bilinguals compared to monolinguals was calculated through between-group contrasts (Bilingual Task vs Monolingual Task).

The second set of group-level models examined the brain-behaviour association between participants’ English proficiency and neural activity for morphological processing. We separated into two models by language group (i.e., a monolingual model and a bilingual model). For each channel, a model was fitted using English vocabulary to predict beta values from the subject-level analysis. The LME formula is “beta ∼ condition*English vocabulary + Age + (1|Participant).” Note that age was added as a covariate. Brain-behaviour associations between participants’ English proficiency and the brain activity for English morphological processing were extracted for each channel.

The third set of group-level models focused on the bilingual group and examined how bilingual proficiency is associated with neural activity for morphological processing. We used Chinese vocabulary and Chinese morphological awareness as the behavioural indicators and fitted two models for each of the behavioural indicators. Here, for each channel, a model was fitted using Chinese vocabulary or morphological awareness to predict beta values, controlling for English vocabulary and age. The LME formula for the models is “beta ∼ condition*Chinese task + English vocabulary + Age + (1|Participant).” Cross-linguistic brain-behaviour associations between participants’ Chinese proficiency and the brain activity for English morphological processing were extracted for each channel.

For each model above, the group-level effects (unstandardized betas) for each contrast or association were plotted on the MNI 152 brain template using the MNI coordinates extracted from the devFold 4-year-old template (described above, see also Fu and Richards, 2021). All group-level effects yielded results with unadjusted p-values and Benjamini-Hochberg FDR-adjusted p-values. The FDR-adjusted p-values are denoted as q henceforth, and they accounted for the number of channels and task comparisons (Huppert et al., 2017, Santosa et al., 2018). Here, our data analyses and interpretations focused on the HbO signals because HbO consists of the majority of the fNIRS signal (HbO 73%–79%; HbR 16%–22% according to a quantification study from Gagnon et al., 2012), and studies have found that HbR signals are not as reliable as they are more susceptible to noise (Hoshi, 2007, Strangman et al., 2002).

## 3 Results

### 3.1 Behavioural task performance

Table 1 reports the means and standard deviations of age, English exposure, and raw and standard scores of the behavioural tasks for each language group. Table 2 reports the correlations between the behavioural scores and fNIRS task accuracy for each language group. Independent samples T-tests were conducted to test group differences. The two groups differed significantly in their amount of English exposure: the monolingual group was exposed to English significantly more than the bilingual group, *t*(153) = −18.99, *p* < .001. Yet, the two language groups did not statistically differ in age, and raw and standard scores of phonological awareness and letter-word identification (*t* values range from −.89 to 1.38, *p* values range from.170 to.806). The two language groups differed in the raw and standard scores of vocabulary. For raw scores, *t*(153) = −5.15, *p* < .001, for standard scores, *t*(153) = −5.17, *p* < .001.

**Table 1:** Behavioural and fNIRS morphological processing task performance (*M*s and *SD*s) for English monolingual and Chinese-English bilingual groups.

	Chinese-English Bilingual	English Monolingual		
	*M(SD)*	*M(SD)*	*T*	*p*
*N*	84	71		
Age (in month)	60.21 (7.1)	61.06 (7.15)	−0.73	0.465
English Exposure %	51.04 (20.68)	96.75 (4.41)	−19.73	< .001
				
*English Task Raw Score*				
Vocabulary (0−240)	99.02 (27.09)	122.14 (26.99)	−5.26	< .001
Phonological Awareness (0−33)	9.77 (5.87)	10.71 (6.46)	−0.91	0.363
Letter-word Identification (0−78)	17.85 (13.45)	16.59 (13.04)	0.59	0.557
				
*Chinese Task Raw Score*				
Vocabulary (0−240)	82.53 (44.73)	/	/	/
Morphological Awareness (0−15)	6.33 (4.08)	/	/	/
*English Task Standard Score*				
Vocabulary (100 ± 15)	99.41 (17.52)	116.38 (21.90)	−5.23	< .001
Phonological Awareness (10 ± 2)	9.42 (2.52)	9.55 (2.76)	−0.31	0.758
Letter-word Identification (100 ± 15)	104.39 (16.20)	100.68 (17.05)	1.38	0.17
				
*fNIRS morphological processing task accuracy*				
Free roots/compounds (0−1)	0.55 (0.14)	0.55 (0.13)	−0.06	0.948
Affixes (0−1)	0.58 (0.16)	0.58 (0.16)	0.04	0.965
Control (0−1)	0.87 (0.16)	0.90 (0.12)	−1.04	0.302

**Table 2:** Correlations between behavioural scores and fNIRS task accuracy by group.

	1	2	3	4
1. Vocabulary	1	0.38***	0.39***	0.45***
2. Phonological Awareness	0.49***	1	0.71***	0.41***
3. Letter-word Identification	0.48***	0.56***	1	0.38**
4. fNIRS overall task accuracy	0.41***	0.45***	0.51***	1

*Note.* Monolingual correlations are above the diagonal, and bilingual correlations are below the diagonal. ** *p* < .01, *** *p* < .001.

To examine whether the fNIRS task accuracies differed by task condition and language group, a 2 (language group: English monolingual and Chinese-English bilingual) * 3 (task conditions: free roots/compounds, affixes, control) mixed ANOVA was performed to predict task accuracy. Results showed a significant main effect of task condition (*F*(2, 306) = 318.52, *p* < .001). Participants showed the highest accuracy in the control condition, *M(SD)* = 88% (14%); followed by the affixes condition, *M(SD)* = 58% (16%); and participants had the lowest accuracy in the free roots/compounds condition *M(SD)* = 54% (14%). Post hoc pairwise analysis with Bonferroni correction revealed that only the two comparisons between experimental conditions and control conditions were significant (both *p* < .001), while affixes accuracy was only marginally higher than the free roots/compounds (*p* = .064). Moreover, neither the group main effect nor the task condition * group interaction was significant (group: *F*(2, 153) = .06, *p* = .803; task condition * group: *F*(2, 306) = .42, *p* = .657).

### 3.2 fNIRS results

#### 3.2.1 Model set 1: Root/compound and affix processing in English monolinguals and Chinese-English bilinguals

**Fig. 4:**
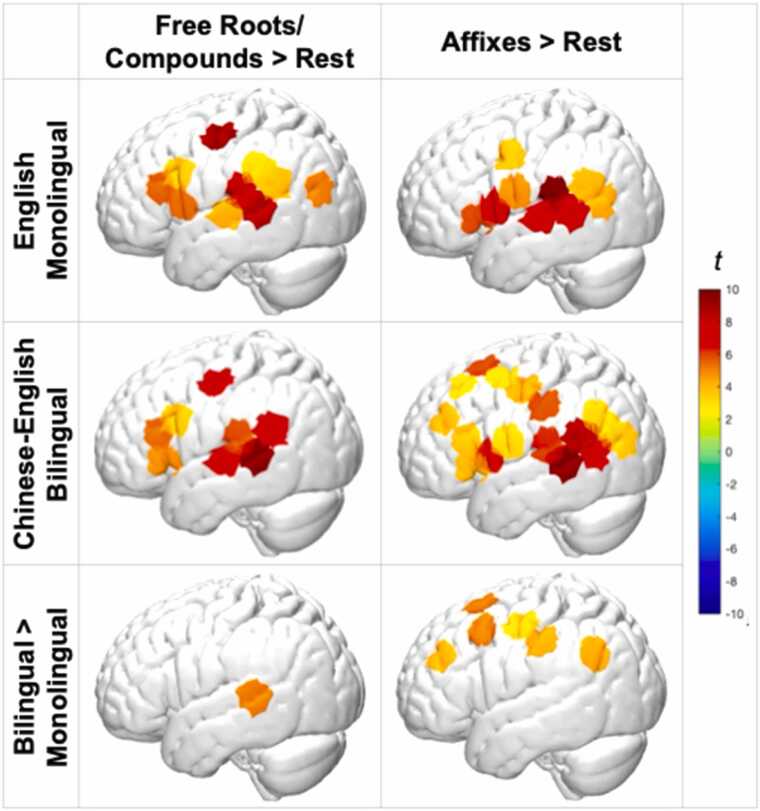
Brain activation in the left hemisphere during Free Roots/Compounds and Affixes conditions (task > rest) and direct contrasts of the two groups (bilingual > monolingual; FDR adjusted at q < 0.001).

The first linear mixed-effects (LME) model examined the brain bases for morphological processing by task condition for each language group. The model used language group and condition to predict brain activation (i.e., beta values from the subject-level general linear models) for each channel. Three sets of contrasts were conducted to reveal the activation for each morphological condition and cross-group activation differences. Results of the left hemisphere were mapped in Fig. 4 with *t* values, and specific LME beta values, t values, and data on the right hemisphere were shown in Supplementary Tables S3-S7 and Figure S2. Significant results used an FDR-adjusted threshold of *q* < .001.

We note that we initially planned to perform Task vs Control contrasts to isolate the specific brain functions unique to morphological processing. However, the two groups showed non-equivalent brain patterns in the baseline control condition: monolinguals engaged both frontal and temporal lobes, while the bilinguals only engaged the auditory temporal regions. This suggests that monolingual participants may not have treated the control blocks as a passive listening task, but rather as a word processing task. We therefore chose to present Task versus Rest activation to represent morphological processing, as this approach allows for more comparable illustrations between the bilingual and monolingual groups. (Please see Supplementary Figure S3 For the activation map in the control condition).

*Task vs Rest.* In the free roots/compounds condition (e.g., bee-bumblebee-frisbee), both groups engaged the left inferior frontal and bilateral superior and middle temporal regions. English monolinguals showed additional left precentral and inferior temporal activation. Chinese bilinguals additionally showed right inferior frontal and middle frontal activation. As for the affix condition (e.g., swimming-walking-something), both groups activated the left inferior frontal, precentral, and bilateral temporal regions. Chinese bilinguals also showed activation in the left superior and middle frontal, and right inferior frontal regions.

*Group differences.* During the free roots/compounds condition, Chinese bilinguals more strongly activated left middle temporal, right superior/middle temporal, and right inferior frontal regions compared to English monolinguals. No channels showed stronger activation in English monolinguals than in Chinese-English bilinguals. During the affixes condition, Chinese bilinguals showed stronger activation of left superior, middle frontal, precentral, postcentral, and right inferior frontal regions. English monolinguals showed stronger activation in right temporal regions compared to Chinese bilinguals.

#### 3.2.2 Model set 2: Brain-behaviour associations with English proficiency in English monolinguals and Chinese-English bilinguals

**Fig. 5:**
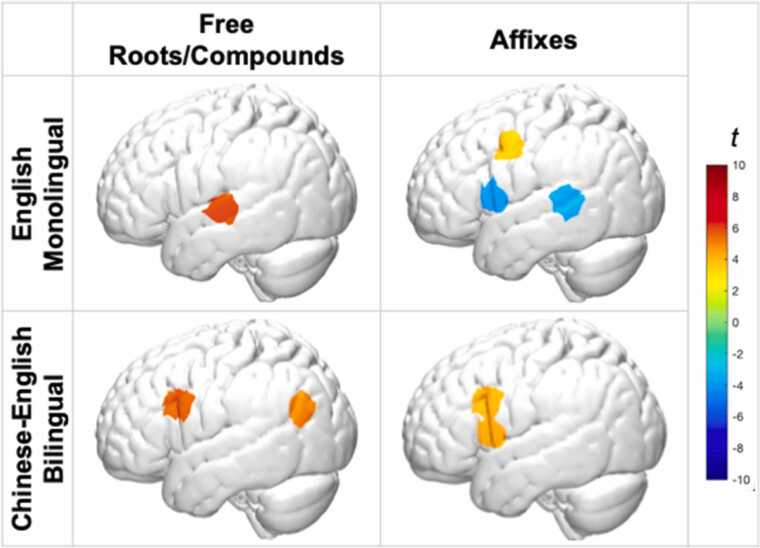
Brain-behaviour associations between English morphological processing and English language skills by Free Roots/Compounds and Affixes/Derivations conditions (*q* < 0.001).

The second set of LME regressions modelled the brain-behaviour associations between English morphological processing and English language skills, as indicated by vocabulary. Two models were conducted for each language group (i.e., a monolingual model and a bilingual model) using vocabulary to predict brain activation for each channel, controlling for age. Results of the left hemisphere by language group were mapped in Fig. 5 with *t* values. Specific LME beta values, t values, and data on the right hemisphere were shown in Supplementary Tables S8-S9 and Figure S4. Significant results used an FDR-adjusted threshold of *q* < .001.

*English Monolinguals.* During the free roots/compounds condition, higher English vocabulary proficiency was associated with stronger left superior/middle temporal (Ch 16; *t* = 6.13), right middle temporal (Ch 36; *t* = 4.59), right inferior frontal (Ch 29, 32; *t*s > 4.21), and right precentral (Ch 31; *t* = 4.73) activation. During the affixes condition, higher English vocabulary proficiency was associated with weaker left inferior frontal (Ch 7; *t* = −4.11), right middle frontal (Ch 25; *t* = −3.86), bilateral middle temporal (Ch 18, 40; *t*s < −3.88) and stronger bilateral precentral (Ch 11, 31; *t* < −4.28) activation.

*Chinese-English Bilinguals.* In both task conditions, higher English vocabulary proficiency was associated with stronger left inferior frontal activation (Roots/compounds: Ch 10; *t* = 5.88; Affixes: Ch 7, 10, *t*s > 4.47). In addition, during the free roots/compounds condition, higher English vocabulary proficiency was also associated with stronger left inferior frontal (Ch 10, *t* = 5.88), middle temporal (Ch 23, *t* = 5.23), right inferior/middle frontal (Ch 25–30; *t*s > 4.6), superior/middle temporal (Ch 34, 37–39, 41, 42; *t*s > 3.71), and precentral activation (Ch 31; *t* = 5.55). No additional associations were significant during the affixes condition.

#### 3.2.3 Model set 3: Brain-behaviour associations with Chinese proficiency in Chinese-English bilinguals

**Fig. 6:**
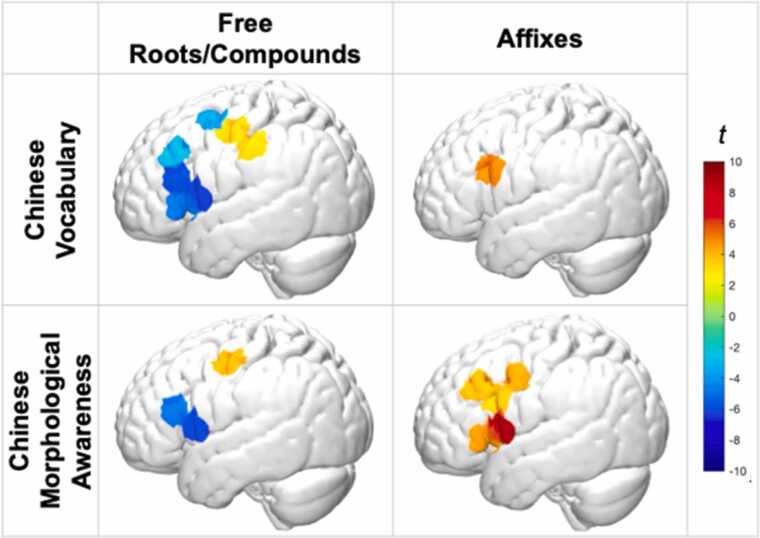
Brain-behaviour associations between English morphological processing and Chinese language skills by Free Roots/Compounds and Affixes/Derivations conditions (*q* < 0.001).

The third set of LME models examined the brain-behaviour associations between English morphological processing and bilinguals’ Chinese language skills, as indicated by Chinese vocabulary and morphological awareness. Two models were conducted for each behavioural task (i.e., a Chinese vocabulary model and a Chinese morphological awareness model) to predict brain activation for each channel, controlling for age and English vocabulary. Results of the left hemisphere by language group were shown in Fig. 6. Specific LME beta values, t values, and data on the right hemisphere were shown in Supplementary Tables S10-S11 and Figure S5. Significant results used an FDR-adjusted threshold of *q* < .001.

*Brain-behaviour associations with Chinese vocabulary.* During the free roots/compounds condition, higher Chinese vocabulary proficiency was associated with weaker left inferior frontal activation (Ch 5, 6, 7, 9; *t*s < −4.4) as well as stronger right middle/inferior frontal (Ch 28, 32; *t*s > 4.17), left postcentral (Ch 15; *t* = 3.97) and bilateral precentral activation (Ch 14, 31; *t*s > 4.02). During the affixes condition, higher Chinese vocabulary proficiency was associated with stronger left inferior frontal activation (Ch 10; *t* = 5.3).

*Brain-behaviour associations with Chinese morphological awareness.* During the free roots/compounds condition, higher Chinese morphological awareness proficiency was associated with weaker left inferior frontal activation (Ch 5, 7; *t*s < −4.85) as well as stronger right middle/inferior frontal (Ch 27, 28; *t*s > 5.46), middle temporal (Ch 34; *t* = 4.26), and bilateral precentral activation (Ch 14, 31; *t*s > 4.7). During the affixes condition, higher Chinese morphological awareness proficiency was associated with stronger left inferior frontal (Ch 6, 7, 10; *t*s > 4.27), middle frontal (Ch 3; *t* = 4.73) and precentral activation (Ch 11; *t* = 5.17).

## 4 Discussion

The overarching question of the current investigation is how early bilingualism influences children’s developing neural architecture for language processing. In light of the theories of bilingual neural development (Chung et al., 2019, Hernandez et al., 2019; Kovelmen & Sun, in press; Yeh et al., 2026), we hypothesized that specific features of bilinguals’ languages may interact and yield unique neural pathways for language compared to monolinguals. Our study focused on morphological word processing in preschool-age children, as it is a spoken language skill emerging from early years and essential to children’s later reading development. Motivated by the structural differences in Chinese and English morphology, we tested Chinese-English bilingual and English monolingual 3-to−5-year-old children with word processing tasks probing different types of morphological structures (i.e., roots/compounds and affixes). The behavioural performance across groups yielded no statistical differences across any task conditions between monolinguals and bilinguals. Our neuroimaging results aligned with the bilingual transfer hypotheses and revealed that experience with Chinese, a language in which the vocabulary consists largely of compounds but few affixes, brings children more automaticity in processing English word roots/compounds, but requires more analytical resources to process the less experienced structure, affixes. Moreover, brain-behaviour associations with participants’ proficiency in each of their languages revealed individual differences in the extent to which diverse language experience influences the developing brain basis for word processing. Together, the findings reveal language-specific impacts on the neural mechanisms of language processing and inform theories of bilingual neural development.

### 4.1 Group differences in the brain basis for the root/compound and affix processing

We hypothesized that Chinese exposure yields a stronger semantic network engagement in bilingual children compared to monolinguals when processing semantically transparent root morphemes. As predicted, during the roots/compounds condition, Chinese-English bilinguals showed stronger activation in semantic-based regions (i.e., left MTG) compared to English monolinguals. This stronger left MTG engagement indicates more specialized and automatic neural processing when accessing semantic information in root morphemes (Weiss et al., 2018). This pattern supports bilingual transfer theories and aligns with prior behavioural evidence that experience with Chinese, a compound-heavy language, affords children stronger sensitivity to root morphemes (Chung et al., 2019, Ramírez et al., 2011).

Our finding provides evidence for an early emerging bilingual transfer effect at the brain level. The transfer effect we identified in compound processing may be specific to this period of spoken language development, before formal literacy instruction begins, as prior work with reader-age children - also using fNIRS - did not show any significant differences in brain activity during English compound processing between Chinese-English bilinguals and English monolinguals (Sun et al., 2022). This prior null effect may suggest no cross-linguistic influence. Yet, it is also likely that as compounding becomes simple and automatic to school-age children, there is a ceiling effect that masks effects from bilingualism. The current evidence supports the latter by showing principled brain differences when preschoolers deal with compounds. Indeed, brain-level bilingual transfer may peak during the preschool years, as once they begin English preschool, children receive less language exposure from their home language (Montrul, 2016). Together, these results highlight the importance of understanding bilingual acquisition as a dynamic process that varies as a function of specific developmental stages and linguistic environments.

Results of the affixes condition also align with our bilingual transfer hypothesis, as shown by the stronger left superior and middle frontal and right inferior frontal activation in Chinese-English bilinguals than in English monolinguals. This pattern of brain activation indicates that higher cognitive demands are required by bilinguals who have less exposure to affix structures. This finding is consistent with prior behavioural evidence showing that Chinese-English bilingual children’s amount of exposure to English is significantly associated with affix but not compound awareness (Sun et al., 2025). Moreover, it aligns with the school-age findings that Chinese-English bilinguals engaged more analytical brain resources (i.e., left MFG and IFG) when accessing English morphological units (Sun et al., 2022, Zhang et al., 2023). In sum, these group-level contrasts offer empirical support for cross-linguistic interactions rooted in structural differences in word morphology at the emergent stage of morphological processing.

The word recognition condition was designed as a passive listening control yet not included in the main analyses due to unexpected group differences. Specifically, while bilinguals showed activation in auditory channels as expected, monolinguals activated additional frontal regions, making task-vs-control contrasts inequivalent across groups. Despite this, these group differences are interesting. For monolinguals, the task may be complex such that children at this young age may find it difficult to switch “off” active word processing during control trials. In contrast, bilinguals’ activation suggests that they may be able to flexibly switch their cognitive strategies between conditions. Indeed, past literature has shown that bilinguals exhibit advantages (at least) in some cognitive tasks (e.g., switching, Bialystok and Viswanathan, 2009, Prior and MacWhinney, 2010). Nonetheless, this is not the focus of the current study, and the interpretations remain tentative. Future research could look into this developmental window and examine whether this pattern is due to bilingual experience.

### 4.2 Individual differences in the brain and English behavioural proficiency

English vocabulary was associated with frontal activation in Chinese-English bilinguals and with temporal activation in English monolinguals. The frontal and temporal involvement is broadly consistent with past research in developmental populations (Jasińska et al., 2021, Skeide et al., 2016). What is novel in the present study is the differential brain regions involved in bilinguals and monolinguals. Past research with English monolingual 5-to−7-year-olds has shown that, although IFG was activated during language tasks, only temporal but not frontal activation was correlated with children’s phonological and semantic task performance (Weiss et al., 2018) and was predictive of longitudinal reading gains (Wang et al., 2020). This literature attributes the lack of brain-behaviour association in the IFG to its delayed specialization compared to temporal networks. Our current monolingual findings aligned with this notion in a younger sample. In contrast, our observation of IFG-behaviour correlation in the bilingual group is particularly noteworthy. Previous studies have reported enhanced IFG engagement in school-age bilinguals compared to monolingual peers during word- and sentence-based language tasks (Arredondo et al., 2018, Jasinska and Petitto, 2013). Therefore, the current brain-behaviour associations may suggest that early bilingualism may enhance IFG specialization for language processing.

Notably, in monolinguals, English vocabulary was positively associated with temporal activation during the roots/compounds condition, but this association was negative during the affixes condition. Prior work with phonological tasks also identified contrasting patterns of brain-behaviour associations. For example, Wang et al. (2018) found a positive association between 5-to−6-year-olds’ reading scores and ventral occipito-temporal (vOT) activation during an onset-matching task, but a negative association during a rhyming task. Their findings suggested that vOT is more sensitive to smaller phonological units (i.e., phoneme onset vs syllable rhyming). Likewise, our results indicate that the middle temporal regions are more sensitive to root-based compound structures, but not the more analytically demanding affix structures. This pattern is consistent with the developmental characteristics of children’s morphological skills, as preschoolers typically demonstrate proficiency with compounds but relatively limited awareness of affixes (Fejzo et al., 2018).

### 4.3 Cross-language brain-behaviour associations between the “English brain” and Chinese proficiency

Both Chinese vocabulary and morphological awareness were significantly associated with increased frontal activation during the English affixes condition and with reduced frontal activation during the English roots/compounds condition, after controlling for age and English vocabulary. These associations further highlight the role of IFG in morphological processing, as previously demonstrated in school-age and adult samples (Gwilliams, 2020, Zou et al., 2016). Importantly, our findings in a preschool sample reveal that early, simultaneous bilingual experience is associated with early specialization of the IFG, as proficiency in Chinese was directly associated with IFG activation during English morphological processing.

The differential association patterns observed across the two morphological task conditions align with the transfer framework. Specifically, experience with Chinese brings ease to compound processing, and thus, after controlling for English proficiency, higher proficiency in Chinese is associated with greater sensitivity to English compound structures, as reflected by reduced IFG demands. In contrast, this pattern runs reversed for affixes: consistent with the explanation that because Chinese lacks affix structures, higher proficiency in Chinese is associated with increased IFG engagement during English affix processing.

### 4.4 Theoretical contributions: The bilingual preschooler brain

Theories of bilingual development generally consider the bilingual child’s brain as an interative, dynamic system influenced by input from both languages (Hernandez et al., 2019, Yeh et al., 2026). The bilingual interactions build upon both person-specific (e.g., developmental dynamics, proficiency) and language-specific factors (e.g., morphological properties, Chung et al., 2019, Kovelman & Sun) to shape the developing brain. Our findings offer novel evidence regarding the particular ways in which early, simultanous bilingual children recruit their neural circuits to process morphological structures. In particular, when analyzing English morphemes, Chinese-English bilingual preschoolers demonstrated a more specialized network by engaging stronger semantic networks when accessing English roots/compounds; in contrast, they adaptively engaged more higher-level cognitive resources when acessing English affixes. These findings are further validated by consistent patterns observed in the brain-behaviour associations with the Chinese behavioural scores.

Our findings also provide insights into building a developmental framework tailored to specific stages of bilingual development. In particular, the roots/compounds findings appear unique to preschool-aged children, as prior studies with school-aged children have primarily revealed effects with affixes (especially derivations), possibly due to their importance in reading (Sun et al., 2023). Bringing these findings together, theoretical frameworks should consider how specific developmental stages interact with language input to shape children’s brains. Moreover, as preschool-aged children sit at the critical developmental transition between spoken language and reading acquisition, neurocognitive models must consider how bilinguals’ neural networks reorganize as children transition to formal reading.

### 4.5 Limitations and future directions

Several limitations should be considered, which merit further investigation. First, although our fNIRS task effectively probed morphological processing and allowed comparison to older children who had been tested with the same task, this task was cognitively challenging for preschool-age participants. This is evidenced by the relatively low overall task accuracy, as well as the brain activity during the control condition in monolinguals, who engaged frontal regions even during passive word control blocks. In contrast, past work with school-age children using the same paradigm (even with more difficult word stimuli) found that children selectively engaged only auditory regions during control trials (Sun et al., 2023). Future work could explore more child-friendly, cognitively accessible methods. Relatedly, the task design and the slow temporal resolution of hemodynamic responses limited our ability to contrast the cognitive processes associated with correct versus incorrect trials, which might show distinct neural bases. Second, while none of the children had entered formal schooling or received systematic reading instruction, some had started learning to read at home, which may have influenced their morphological development. The current investigation focused on the role of bilingualism and thus matched reading proficiency between the two groups. Future work could further explore the impact of early reading experience on the neurocognitive profiles underlying morphological processing.

In summary, the current study examined the effects of early bilingual experience on the neural basis of language in preschool children, with a particular focus on morphological word processing. Our results showed that preschool-age children exhibited specialized brain networks for segmenting meaningful units from words. Notably, our bilingual findings support the transfer framework, indicating that the young bilingual brain shows transfer with concomitant sensitivity to shared morphological structures from one language to the other. These results highlight the plasticity in neural organization during the transitional period between oral-only language and reading acquisition by revealing how emerging brain functions for language are shaped by bilingual experience.

## CRediT authorship contribution statement

**Janet F. Werker:** Writing – review & editing, Writing – original draft, Supervision, Resources, Investigation, Funding acquisition, Conceptualization. **Xin Sun:** Writing – review & editing, Writing – original draft, Visualization, Validation, Supervision, Resources, Project administration, Methodology, Investigation, Funding acquisition, Formal analysis, Data curation, Conceptualization.

## Funding

This research was supported by a Partnership Grant (ID: 895-2020-1004) from the Social Sciences and Humanities Research Council awarded to J. F. Werker, and a Strategic Hiring Scheme grant (ID: P0059372) from The Hong Kong Polytechnic University awarded to X. Sun.

## Declaration of Competing Interest

There are no known competing interests to declare.

## Data Availability

Data will be made available on request.

## Footnotes

Appendix A Supplementary data associated with this article can be found in the online version at doi:10.1016/j.dcn.2026.101783.
