# Supplementary material for: Meanings within words: How Chinese bilingualism shapes the neural basis of English morphological processing in preschoolers
Source: Dev Cogn Neurosci. 2026 Jul 9;80:101783. doi: 10.1016/j.dcn.2026.101783 (PMC13382041; doi:10.1016/j.dcn.2026.101783)
Supplement: Supplementary file 1 — Supplementary material [file mmc1.docx]

***Supplementary Materials to manuscript***

**English morphological processing in the preschooler brain: A study with Chinese-English bilinguals and English monolinguals**

**Table S1**

**Word Stimuli, their characteristics, age of acquisition, and block design**

|  |  |  | Number of… | | | AELP AOA | | Kuperman |
| --- | --- | --- | --- | --- | --- | --- | --- | --- |
|  |  | Stim | Phon. | Syll. | Morph. | *M* | *SD* | *M* |
| Block1 | 1 | Human | 6 | 2 | 1 | 4.83 | 1.76 | 4.83 |
| Comp |  | Woman | 5 | 2 | 1 | 4.95 | 2.19 | 4.95 |
|  |  | Lemon | 5 | 2 | 1 | 4.74 | 1.73 | 4.74 |
|  | 2 | Pen | 3 | 1 | 1 | 5.11 | 1.29 | 5.11 |
|  |  | Pencil | 6 | 2 | 1 | 4.06 | 1.8 | 4.06 |
|  |  | Penguin | 7 | 2 | 1 | 5.68 | 2.4 | 5.68 |
|  | 3 | Win | 3 | 1 | 1 | 4.22 | 1.31 | 4.22 |
|  |  | Window | 5 | 2 | 1 | 4.74 | 2.47 | 4.74 |
|  |  | Winner | 4 | 2 | 2 |  |  | 5.55 |
|  | 4 | Woman | 5 | 2 | 1 | 4.95 | 2.19 | 4.95 |
|  |  | Cinnamon | 7 | 3 | 1 | 5.37 | 3.34 | 4.72 |
|  |  | Mailman | 6 | 2 | 2 |  |  | N/A |
|  |  |  |  |  |  |  |  |  |
| Block2 | 1 | Lady | 4 | 2 | 1 | 3.68 | 1.63 | 3.68 |
| Ctrl |  | Tongue | 3 | 1 | 1 | 4.47 | 2.87 | 4.47 |
|  | 2 | Welcome | 6 | 2 | 1 | 4.06 | 1.21 | 4.06 |
|  |  | Cartoon | 6 | 2 | 1 | 3.11 | 1.32 | 3.11 |
|  | 3 | Breakfast | 8 | 2 | 2 | 3.47 | 1.81 | 3.47 |
|  |  | Jacket | 5 | 2 | 1 | 3.95 | 2.09 | 3.95 |
|  | 4 | Monkey | 5 | 2 | 1 | 4.21 | 1.4 | 4.21 |
|  |  | After | 4 | 2 | 1 |  |  | 6 |
|  |  |  |  |  |  |  |  |  |
| Block3 | 1 | Birthday | 6 | 2 | 2 |  |  | 2.85 |
| Comp |  | Stay | 3 | 1 | 1 |  |  | 4.22 |
|  |  | Everyday | 6 | 3 | 2 | 5.05 | 1.72 | 5.05 |
|  | 2 | Fish | 3 | 1 | 1 | 4.05 | 1.61 | 4.05 |
|  |  | Selfish | 6 | 2 | 2 | 5.26 | 1.76 | 5.26 |
|  |  | Goldfish | 7 | 2 | 2 | 4.5 | 3.43 | 4.5 |
|  | 3 | Bee | 2 | 1 | 1 | 5 | 1.97 | 5 |
|  |  | Bumblebee | 8 | 3 | 2 | 4.11 | 1.37 | 4.11 |
|  |  | Frisbee | 5 | 2 | 1 |  |  | 5.5 |
|  | 4 | End | 3 | 1 | 1 | 4.28 | 2.61 | 4.28 |
|  |  | Weekend | 6 | 2 | 2 |  |  | 4.79 |
|  |  | Friend | 5 | 1 | 1 | 3.57 | 1.43 | 3.57 |
|  |  |  |  |  |  |  |  |  |
| Block4 | 1 | Smiling | 6 | 2 | 2 |  |  |  |
| Affix |  | Wing | 3 | 1 | 1 | 4.79 | 1.84 | 4.79 |
|  |  | Talking | 6 | 2 | 2 |  |  | N/A |
|  | 2 | Eating | 5 | 2 | 2 |  |  | N/A |
|  |  | Ring | 3 | 1 | 1 | 4.53 | 1.87 | 4.53 |
|  |  | Looking | 6 | 2 | 2 |  |  | N/A |
|  | 3 | Running | 5 | 2 | 2 | NULL | NULL | N/A |
|  |  | Riding | 5 | 2 | 2 | NULL | NULL | N/A |
|  |  | King | 3 | 1 | 1 | 5.42 | 2.63 | 5.42 |
|  | 4 | Reading | 5 | 2 | 2 | NULL | NULL | N/A |
|  |  | Driving | 6 | 2 | 2 | NULL | NULL | N/A |
|  |  | Swing | 4 | 1 | 1 | 4.16 | 2.03 | 4.16 |
|  |  |  |  |  |  |  |  |  |
| Block5 | 1 | Father | 5 | 2 | 1 | 4.11 | 1.76 | 4.11 |
| Ctrl |  | Balloon | 5 | 2 | 1 | 4.37 | 1.95 | 4.37 |
|  | 2 | Napkin | 6 | 2 | 1 | 4.79 | 2.51 | 4.79 |
|  |  | Children | 7 | 2 | 2 | 4.095 | 1.14 | 4.1 |
|  | 3 | Drink | 5 | 1 | 1 | 3.47 | 1.31 | 3.47 |
|  |  | Marker | 5 | 2 | 1 |  |  | 4.89 |
|  | 4 | Groud | 5 | 1 | 1 | 4.89 | 2.3 | 4.89 |
|  |  | Story | 5 | 2 | 1 | 3.89 | 1.59 | 3.89 |
|  |  |  |  |  |  |  |  |  |
| Block6 | 1 | Singer | 4 | 2 | 2 |  |  | 5.06 |
| Affix |  | Reader | 4 | 2 | 2 |  |  | 5.37 |
|  |  | Corner | 6 | 2 | 1 | 5.21 | 2.07 | 5.21 |
|  | 2 | Firefighter | 9 | 3 | 3 |  |  | 5.16 |
|  |  | Another | 6 | 3 | 1 | 5.049 | 1.79 | 5.05 |
|  |  | Winner | 4 | 2 | 2 |  |  | 5.55 |
|  | 3 | Rider | 5 | 2 | 2 | 6.37 | 2.03 | 6.37 |
|  |  | Stranger | 8 | 2 | 2 | 4.53 | 1.93 | 4.53 |
|  |  | Water | 5 | 2 | 1 | 2.37 | 0.76 | 5.6 |
|  | 4 | Painter | 5 | 2 | 2 |  |  | 4.38 |
|  |  | Winter | 6 | 2 | 1 | 4.38 | 1.66 | 6 |
|  |  | Dancer | 5 | 2 | 2 |  |  | N/A |
|  |  |  |  |  |  |  |  |  |
| Block7 | 1 | Blackboard | 7 | 2 | 2 |  |  | 5.42 |
| Ctrl |  | Shampoo | 5 | 2 | 1 | 5.39 | 2.7 | 5.39 |
|  | 2 | Camping | 6 | 2 | 2 |  |  | 5.33 |
|  |  | Needle | 4 | 1 | 1 | 5.32 | 1.53 | 5.32 |
|  | 3 | Bottom | 5 | 2 | 1 | 5.28 | 2.08 | 5.28 |
|  |  | Accident | 8 | 3 | 1 | 5.3 | 2.18 | 5.3 |
|  | 4 | Nobody | 6 | 3 | 2 | 5.252 | 2.07 | 5.25 |
|  |  | Stain | 4 | 1 | 1 | 5.24 | 1.41 | 5.24 |
|  |  |  |  |  |  |  |  |  |
|  |  |  |  |  |  |  |  |  |
| Block8 | 1 | Bee | 2 | 1 | 1 | 5 | 1.97 | 5 |
| Comp |  | Beehive | 5 | 2 | 2 | 7.47 | 5.05 | 7.47 |
|  |  | Beach | 3 | 1 | 1 | 4.8 | 1.54 | 4.8 |
|  | 2 | Teacup | 4 | 2 | 2 |  |  | 5.39 |
|  |  | T-rex | 4 | 1 | 1 |  |  | N/A |
|  |  | Teaspoon | 6 | 2 | 2 | 5.26 | 2.45 | 5.26 |
|  | 3 | Something | 6 | 2 | 2 | 5.04944 | 2.15 | 5.05 |
|  |  | Someone | 5 | 2 | 2 |  |  | 4.92 |
|  |  | Summer | 5 | 2 | 1 | 4.33 | 1.68 | 4.33 |
|  | 4 | Sea | 2 | 1 | 1 |  |  | 4.74 |
|  |  | Seat | 3 | 1 | 1 | 4.58 | 1.92 | 4.58 |
|  |  | Seagull | 5 | 2 | 2 | 5.42 | 2.01 | 5.42 |
|  |  |  |  |  |  |  |  |  |
|  |  |  |  |  |  |  |  |  |
| Block9 | 1 | Helping | 6 | 2 | 2 |  |  | N/A |
| Affix |  | Kicking | 6 | 2 | 2 |  |  | N/A |
|  |  | Bring | 4 | 1 | 1 | 4.42 | 2.46 | 4.42 |
|  | 2 | Throwing | 7 | 2 | 2 |  |  | N/A |
|  |  | Sing | 3 | 1 | 1 | 3.47 | 1.31 | 3.47 |
|  |  | Playing | 6 | 2 | 2 |  |  | N/A |
|  | 3 | Dancing | 6 | 2 | 2 |  |  | 4.09 |
|  |  | Singing | 6 | 2 | 2 |  |  | N/A |
|  |  | Morning | 6 | 2 | 1 | 4 | 1.45 | 4 |
|  | 4 | Swimming | 7 | 2 | 2 |  |  | 4.58 |
|  |  | Something | 6 | 2 | 2 | 5.0494 | 2.15 | 5.05 |
|  |  | Walking | 6 | 2 | 2 |  |  | N/A |
|  |  |  |  |  |  |  |  | N/A |
| Block10 | 1 | Eater | 4 | 2 | 2 |  |  | 5.94 |
| Affix |  | Finger | 6 | 2 | 1 | 3.43 | 2.2 | 3.43 |
|  |  | Talker | 5 | 2 | 2 |  |  | 5.74 |
|  | 2 | Winter | 6 | 2 | 1 | 4.38 | 1.66 | 4.38 |
|  |  | Diver | 4 | 2 | 2 |  |  | 6.89 |
|  |  | Shoulder | 6 | 2 | 1 | 4.5 | 1.42 | 4.5 |
|  | 3 | Farmer | 5 | 2 | 2 |  |  | 4.74 |
|  |  | Soccer | 5 | 2 | 1 | 5.89 | 4 | 5.89 |
|  |  | Brother | 6 | 2 | 1 | 3.63 | 1.54 | 3.63 |
|  | 4 | Swimmer | 6 | 2 | 2 |  |  | 5.11 |
|  |  | Player | 5 | 2 | 2 | 6.89 | 3.27 | 6.89 |
|  |  | Summer | 5 | 2 | 1 | 4.33 | 1.68 | 4.33 |
|  |  |  |  |  |  |  |  |  |
| Block11 | 1 | Green | 4 | 1 | 1 |  |  | 3.79 |
| Ctrl |  | Dentist | 7 | 2 | 1 | 5.22 | 2.51 | 5.22 |
|  | 2 | Magic | 5 | 2 | 1 | 5.52 | 1.94 | 5.52 |
|  |  | Raincoat | 6 | 2 | 2 |  |  | 5.5 |
|  | 3 | Squeeze | 5 | 1 | 1 | 5.42 | 2.17 | 5.42 |
|  |  | Proud | 4 | 1 | 1 | 5.44 | 1.58 | 5.44 |
|  | 4 | Muddy | 4 | 2 | 1 |  |  | 5.42 |
|  |  | Lipstick | 7 | 2 | 2 | 5.42 | 2.59 | 5.42 |
|  |  |  |  |  |  |  |  |  |
| Block12 | 1 | Motorcycle | 10 | 4 | 2 | 5.05 | 1.54 | 5.05 |
| Comp |  | Bicycle | 7 | 3 | 2 | 4.26 | 1.45 | 4.26 |
|  |  | Uncle | 4 | 2 | 1 |  |  | 4.47 |
|  | 2 | Mama | 4 | 2 | 1 | 1.89 | 1.29 | 1.89 |
|  |  | Pajama | 6 | 3 | 1 |  |  | 4.27 |
|  |  | Grandma | 6 | 2 | 2 |  |  | 2.58 |
|  | 3 | Sand | 4 | 1 | 1 | 4.63 | 2.31 | 4.63 |
|  |  | Sandbox | 6 | 2 | 2 |  |  | 4.14 |
|  |  | Sandwich | 7 | 2 | 1 | 4.79 | 1.93 | 4.79 |
|  | 4 | Eye | 1 | 1 | 1 | 3.75 | 1.71 | 3.75 |
|  |  | Island | 5 | 2 | 1 | 7.41 | 2.81 | 7.41 |
|  |  | Eyeball | 5 | 2 | 2 |  |  | 3.83 |

**Table S2**

**Channel MNI Coordinates (adopted from devFold, Xu & Richards, 2021)**

| S | D | X | Y | Z | Landmark | Specificity | CH | S-D |
| --- | --- | --- | --- | --- | --- | --- | --- | --- |
| AF3 | F5 | -42 | 56 | 19 | Frontal_Mid_2_L | 70.6 | 1 | S1-D2 |
| FC5 | F5 | -59 | 30 | 19 | Frontal_Inf_Tri_L | 58.9 | 10 | S5-D2 |
| FC5 | FC3 | -60 | 17 | 34 | Precentral_L | 46.0 | 11 | S5-D4 |
| FC5 | FT7 | -67 | 13 | 8 | L Superior Temporal G | 36.7 | 12 | S5-D5 |
| FC5 | C5 | -68 | 1 | 21 | Brain_Outside | 39.2 | 13 | S5-D6 |
| C3 | FC3 | -57 | 3 | 48 | L Precentral Gyrus | 44.0 | 14 | S6-D4 |
| C3 | C5 | -68 | -12 | 38 | Brain_Outside | 46.7 | 15 | S6-D6 |
| T7 | FT7 | -70 | -4 | -9 | Temporal_Mid_L | 56.9 | 16 | S7-D5 |
| T7 | C5 | -74 | -15 | 6 | Temporal_Sup_L | 46.1 | 17 | S7-D6 |
| T7 | TP7 | -72 | -28 | -6 | Temporal_Mid_L | 63.4 | 18 | S7-D7 |
| CP5 | C5 | -72 | -27 | 25 | Temporal_Sup_L | 52.7 | 19 | S8-D6 |
| F3 | F1 | -33 | 47 | 42 | Frontal_Mid_2_L | 67.0 | 2 | S2-D1 |
| CP5 | TP7 | -71 | -41 | 13 | Temporal_Mid_L | 59.0 | 20 | S8-D7 |
| CP5 | P5 | -66 | -55 | 29 | Temporal_Mid_L | 46.8 | 21 | S8-D8 |
| P7 | TP7 | -67 | -52 | 2 | Temporal_Inf_L | 62.6 | 22 | S9-D7 |
| P7 | P5 | -62 | -65 | 18 | Temporal_Mid_L | 73.0 | 23 | S9-D8 |
| P7 | PO7 | -56 | -76 | 9 | Temporal_Inf_L | 37.0 | 24 | S9-D16 |
| F4 | F6 | 51 | 41 | 28 | Frontal_Mid_2_R | 68.0 | 25 | S10-D9 |
| F4 | FC4 | 48 | 28 | 43 | Frontal_Mid_2_R | 74.7 | 26 | S10-D11 |
| F8 | F6 | 58 | 37 | 8 | Frontal_Inf_Tri_R | 77.5 | 27 | S11-D9 |
| F8 | F10 | 59 | 33 | -12 | Frontal_Inf_Orb_2_R | 42.1 | 28 | S11-D10 |
| F8 | FT8 | 63 | 23 | -4 | Frontal_Inf_Orb_2_R | 35.4 | 29 | S11-D12 |
| F3 | F5 | -49 | 44 | 26 | Frontal_Mid_2_L | 61.1 | 3 | S2-D2 |
| FC6 | F6 | 60 | 25 | 20 | Frontal_Inf_Tri_R | 66.0 | 30 | S12-D9 |
| FC6 | FC4 | 59 | 15 | 35 | Brain_Outside | 52.7 | 31 | S12-D11 |
| FC6 | FT8 | 66 | 11 | 10 | Frontal_Inf_Oper_R | 46.8 | 32 | S12-D12 |
| FC6 | C6 | 67 | -2 | 22 | Brain_Outside | 49.5 | 33 | S12-D13 |
| FT10 | F10 | 64 | 12 | -36 | R Middle Temporal G | 52.2 | 34 | S13-D10 |
| FT10 | FT8 | 66 | 6 | -19 | Temporal_Pole_Mid_R | 36.9 | 35 | S13-D10 |
| T8 | FT8 | 69 | -7 | -7 | Temporal_Mid_R | 80.8 | 36 | S14-D12 |
| T8 | C6 | 71 | -18 | 9 | Temporal_Mid_R | 71.0 | 37 | S14-D13 |
| T8 | TP8 | 70 | -33 | -4 | Temporal_Mid_R | 74.0 | 38 | S14-D14 |
| CP6 | C6 | 69 | -30 | 28 | Brain_Outside | 50.9 | 39 | S15-D13 |
| F3 | FC3 | -49 | 31 | 41 | Frontal_Mid_2_L | 77.4 | 4 | S2-D4 |
| CP6 | TP8 | 68 | -44 | 14 | Temporal_Mid_R | 81.2 | 40 | S15-D14 |
| CP6 | P6 | 62 | -58 | 32 | Brain_Outside | 45.3 | 41 | S15-D15 |
| P8 | TP8 | 64 | -57 | 3 | Temporal_Mid_R | 54.9 | 42 | S16-D14 |
| P8 | P6 | 57 | -70 | 20 | Temporal_Mid_R | 57.8 | 43 | S16-D15 |
| F7 | F5 | -55 | 41 | 7 | Frontal_Inf_Tri_L | 72.1 | 5 | S3-D2 |
| F7 | F9 | -55 | 36 | -13 | Frontal_Inf_Orb_2_L | 36.7 | 6 | S3-D3 |
| F7 | FT7 | -61 | 26 | -5 | Temporal_Pole_Sup_L | 34.4 | 7 | S3-D5 |
| FC1 | F1 | -27 | 33 | 56 | Frontal_Sup_2_L | 73.3 | 8 | S4-D1 |
| FC1 | FC3 | -43 | 19 | 54 | Frontal_Mid_2_L | 67.9 | 9 | S4-D4 |

**Table S3**

*Chinese-English Bilinguals’ Activations during the Free Roots/Compounds Condition Relative to Rest*

| ***Hem.-Ch#*** | ***Source-Detector*** | ***Region*** | ***β*** | ***se*** | ***t-stat*** | ***p*** | ***q*** |
| --- | --- | --- | --- | --- | --- | --- | --- |
| L-18 | S7-D7 | Middle Temporal | 388.29 | 23.51 | 16.52 | <0.001 | <0.001 |
| L-14 | S6-D4 | Precentral | 256.68 | 31.19 | 8.23 | <0.001 | <0.001 |
| L-20 | S8-D7 | Middle Temporal | 141.21 | 20.28 | 6.96 | <0.001 | <0.001 |
| L-16 | S7-D5 | Middle Temporal | 159.65 | 23.27 | 6.86 | <0.001 | <0.001 |
| L-17 | S7-D6 | Superior Temporal | 124.46 | 20.71 | 6.01 | <0.001 | <0.001 |
| L-5 | S3-D2 | Inferior Frontal | 116.51 | 20.50 | 5.68 | <0.001 | <0.001 |
| L-6 | S3-D3 | Inferior Frontal | 130.28 | 26.72 | 4.88 | <0.001 | <0.001 |
| L-10 | S5-D2 | Inferior Frontal | 94.82 | 23.15 | 4.10 | <0.001 | <0.001 |
| R-38 | S14-D14 | Middle Temporal | 307.34 | 27.52 | 11.17 | <0.001 | <0.001 |
| R-28 | S11-D10 | Inferior Frontal | 207.48 | 26.98 | 7.69 | <0.001 | <0.001 |
| R-35 | S13-D12 | Middle Temporal | 246.73 | 32.06 | 7.70 | <0.001 | <0.001 |
| R-37 | S14-D13 | Middle Temporal | 146.89 | 21.48 | 6.84 | <0.001 | <0.001 |
| R-36 | S14-D12 | Middle Temporal | 153.04 | 23.28 | 6.57 | <0.001 | <0.001 |
| R-26 | S10-D11 | Middle Frontal | 160.78 | 26.98 | 5.96 | <0.001 | <0.001 |

**Table S4**

*English Monolinguals’ Activations during the Free Roots/Compounds Condition Relative to Rest*

| **Hem.-Ch#** | **Source-Detector** | **Region** | ***β*** | ***se*** | ***t*-stat** | ***p*** | ***q*** |
| --- | --- | --- | --- | --- | --- | --- | --- |
| L-14 | S6-D4 | Precentral | 208.66 | 21.78 | 9.58 | <0.001 | <0.001 |
| L-18 | S7-D7 | Middle Temporal | 208.43 | 23.31 | 8.94 | <0.001 | <0.001 |
| L-17 | S7-D6 | Superior Temporal | 156.60 | 18.47 | 8.48 | <0.001 | <0.001 |
| L-5 | S3-D2 | Inferior Frontal | 111.15 | 19.05 | 5.83 | <0.001 | <0.001 |
| L-7 | S3-D5 | Inferior Frontal | 144.40 | 26.27 | 5.50 | <0.001 | <0.001 |
| L-24 | S9-D16 | Inferior Temporal | 120.37 | 23.01 | 5.23 | <0.001 | <0.001 |
| L-16 | S7-D5 | Middle Temporal | 97.12 | 21.54 | 4.51 | <0.001 | <0.001 |
| L-20 | S8-D7 | Middle Temporal | 79.83 | 18.96 | 4.21 | <0.001 | <0.001 |
| L-10 | S5-D2 | Inferior Frontal | 85.38 | 20.77 | 4.11 | <0.001 | <0.001 |
| L-19 | S8-D6 | Superior Temporal | 88.33 | 23.59 | 3.74 | <0.001 | <0.001 |
| R-36 | S14-D12 | Middle Temporal | 113.32 | 22.97 | 4.93 | <0.001 | <0.001 |
| R-38 | S14-D14 | Middle Temporal | 111.52 | 30.14 | 3.70 | <0.001 | <0.001 |

**Table S5**

*Chinese-English Bilinguals’ Activations during the Affixes Condition Relative to Rest*

| **Hem.-Ch#** | **Source-Detector** | **Region** | ***β*** | ***se*** | ***t*-stat** | ***p*** | ***q*** |
| --- | --- | --- | --- | --- | --- | --- | --- |
| L-18 | S7-D7 | Middle Temporal | 232.54 | 22.57 | 10.30 | <0.001 | <0.001 |
| L-20 | S8-D7 | Middle Temporal | 177.13 | 19.67 | 9.00 | <0.001 | <0.001 |
| L-22 | S9-D7 | Inferior Temporal | 178.06 | 23.96 | 7.43 | <0.001 | <0.001 |
| L-7 | S3-D5 | Inferior Frontal | 175.38 | 26.96 | 6.50 | <0.001 | <0.001 |
| L-17 | S7-D6 | Superior Temporal | 126.21 | 19.78 | 6.38 | <0.001 | <0.001 |
| L-8 | S4-D1 | Superior Frontal | 156.83 | 25.24 | 6.21 | <0.001 | <0.001 |
| L-15 | S6-D6 | Postcentral | 132.10 | 21.51 | 6.14 | <0.001 | <0.001 |
| L-14 | S6-D4 | Precentral | 142.06 | 30.03 | 4.73 | <0.001 | <0.001 |
| L-1 | S1-D2 | Middle Frontal | 112.50 | 25.04 | 4.49 | <0.001 | <0.001 |
| L-5 | S3-D2 | Inferior Frontal | 83.13 | 19.66 | 4.23 | <0.001 | <0.001 |
| L-6 | S3-D3 | Inferior Frontal | 107.65 | 25.95 | 4.15 | <0.001 | <0.001 |
| L-21 | S8-D8 | Middle Temporal | 86.21 | 22.61 | 3.81 | <0.001 | <0.001 |
| L-24 | S9-D16 | Inferior Temporal | 90.65 | 24.14 | 3.75 | <0.001 | <0.001 |
| L-12 | S5-D5 | Superior Temporal | 70.81 | 19.64 | 3.61 | <0.001 | <0.001 |
| L-9 | S4-D4 | Middle Frontal | 71.14 | 19.79 | 3.60 | <0.001 | <0.001 |
| L-23 | S9-D8 | Middle Temporal | 62.15 | 17.76 | 3.50 | <0.001 | <0.001 |
| L-2 | S2-D1 | Middle Frontal | 59.57 | 17.30 | 3.44 | <0.001 | <0.001 |
| R-38 | S14-D14 | Middle Temporal | 323.19 | 26.87 | 12.03 | <0.001 | <0.001 |
| R-36 | S14-D12 | Middle Temporal | 214.16 | 22.44 | 9.54 | <0.001 | <0.001 |
| R-37 | S14-D13 | Middle Temporal | 184.05 | 20.93 | 8.79 | <0.001 | <0.001 |
| R-40 | S15-D14 | Middle Temporal | 155.27 | 24.12 | 6.44 | <0.001 | <0.001 |
| R-35 | S13-D12 | Middle Temporal | 188.80 | 30.63 | 6.16 | <0.001 | <0.001 |
| R-42 | S16-D14 | Middle Temporal | 127.92 | 23.75 | 5.39 | <0.001 | <0.001 |
| R-41 | S15-D15 | Middle Temporal | 99.07 | 25.11 | 3.95 | <0.001 | <0.001 |
| R-29 | S11-D12 | Inferior Frontal | 100.47 | 26.47 | 3.80 | <0.001 | <0.001 |
| R-33 | S12-D13 | Inferior Frontal | 98.50 | 26.74 | 3.68 | <0.001 | <0.001 |

**Table S6**

*English Monolinguals’ Activations during the Affixes Condition Relative to Rest*

| **Hem.-Ch#** | **Source-Detector** | **Region** | ***β*** | ***se*** | ***t*-stat** | ***p*** | ***q*** |
| --- | --- | --- | --- | --- | --- | --- | --- |
| L-17 | S7-D6 | Superior Temporal | 197.95 | 18.03 | 10.98 | <0.001 | <0.001 |
| L-7 | S3-D5 | Inferior Frontal | 197.48 | 25.16 | 7.85 | <0.001 | <0.001 |
| L-18 | S7-D7 | Middle Temporal | 170.74 | 22.67 | 7.53 | <0.001 | <0.001 |
| L-16 | S7-D5 | Middle Temporal | 144.99 | 20.97 | 6.91 | <0.001 | <0.001 |
| L-6 | S3-D3 | Inferior Frontal | 160.53 | 26.61 | 6.03 | <0.001 | <0.001 |
| L-12 | S5-D5 | Superior Temporal | 87.30 | 17.38 | 5.02 | <0.001 | <0.001 |
| L-22 | S9-D7 | Inferior Temporal | 102.00 | 22.44 | 4.54 | <0.001 | <0.001 |
| L-20 | S8-D7 | Middle Temporal | 80.80 | 18.40 | 4.39 | <0.001 | <0.001 |
| L-11 | S5-D4 | Precentral | 81.71 | 18.75 | 4.36 | <0.001 | <0.001 |
| R-36 | S14-D12 | Middle Temporal | 257.74 | 22.04 | 11.70 | <0.001 | <0.001 |
| R-38 | S14-D14 | Middle Temporal | 230.86 | 29.24 | 7.90 | <0.001 | <0.001 |
| R-37 | S14-D13 | Middle Temporal | 145.69 | 20.52 | 7.10 | <0.001 | <0.001 |
| R-40 | S15-D14 | Middle Temporal | 95.35 | 21.94 | 4.35 | <0.001 | <0.001 |

**Table S7**

*Chinese-English Bilingual Versus English Monolingual Activation Subtraction*

| **Hem.-Ch#** | **Source-Detector** | **Region** | ***β*** | ***se*** | ***t-stat*** | ***p*** | ***q*** |
| --- | --- | --- | --- | --- | --- | --- | --- |
| *Free roots/compounds condition* | | | | | | | |
| L-18 | S7-D7 | Middle Temporal | 179.85 | 33.10 | 5.43 | <0.001 | <0.001 |
| R-35 | S13-D12 | Middle Temporal | 393.25 | 42.17 | 9.32 | <0.001 | <0.001 |
| R-41 | S15-D15 | Middle Temporal | 309.16 | 36.63 | 8.44 | <0.001 | <0.001 |
| R-39 | S15-D13 | Superior Temporal | 300.24 | 41.49 | 7.24 | <0.001 | <0.001 |
| R-42 | S16-D14 | Middle Temporal | 199.09 | 34.22 | 5.82 | <0.001 | <0.001 |
| R-38 | S14-D14 | Middle Temporal | 195.82 | 40.81 | 4.80 | <0.001 | <0.001 |
| R-33 | S12-D13 | Inferior Frontal | 160.88 | 39.22 | 4.10 | <0.001 | <0.001 |
|  | | | | | | | |
| *Affixes condition* | | | | | | | |
| L-8 | S4-D1 | Superior Frontal | 186.93 | 33.42 | 5.59 | <0.001 | <0.001 |
| L-4 | S2-D4 | Middle Frontal | 215.73 | 39.20 | 5.50 | <0.001 | <0.001 |
| L-15 | S6-D6 | Postcentral | 141.69 | 29.39 | 4.82 | <0.001 | <0.001 |
| L-21 | S8-D8 | Middle Temporal | 148.50 | 31.05 | 4.78 | <0.001 | <0.001 |
| L-1 | S1-D2 | Middle Frontal | 162.18 | 35.18 | 4.61 | <0.001 | <0.001 |
| L-14 | S6-D4 | Precentral | 143.09 | 36.73 | 3.90 | <0.001 | <0.001 |
| R-30 | S12-D9 | Inferior Frontal | 189.01 | 36.90 | 5.12 | <0.001 | <0.001 |
| R-27 | S11-D9 | Inferior Frontal | 108.83 | 26.36 | 4.13 | <0.001 | <0.001 |
| R-39 | S15-D13 | Superior Temporal | -161.78 | 40.34 | -4.01 | <0.001 | <0.001 |
| R-41 | S15-D15 | Middle Temporal | 139.03 | 35.55 | 3.91 | <0.001 | <0.001 |

**Table S8**

*Chinese-English Bilinguals’ Brain Behavior Association with English Vocabulary*

| **Hem.-Ch#** | **Source-Detector** | **Region** | ***β*** | ***se*** | ***t*-stat** | ***p*** | ***q*** |
| --- | --- | --- | --- | --- | --- | --- | --- |
| *Free roots/compounds condition* | | | | | | | |
| L-10 | S5-D2 | Inferior Frontal | 5.54 | 0.94 | 5.88 | <0.001 | <0.001 |
| L-23 | S9-D8 | Middle Temporal | 3.78 | 0.72 | 5.23 | <0.001 | <0.001 |
| R-38 | S14-D14 | Middle Temporal | 8.72 | 1.11 | 7.84 | <0.001 | <0.001 |
| R-26 | S10-D11 | Middle Frontal | 8.62 | 1.13 | 7.65 | <0.001 | <0.001 |
| R-25 | S10-D9 | Middle Frontal | 6.04 | 0.84 | 7.14 | <0.001 | <0.001 |
| R-41 | S15-D15 | Middle Temporal | 7.44 | 1.04 | 7.16 | <0.001 | <0.001 |
| R-28 | S11-D10 | Inferior Frontal | 6.42 | 1.02 | 6.28 | <0.001 | <0.001 |
| R-31 | S12-D11 | Precentral | 5.48 | 0.99 | 5.55 | <0.001 | <0.001 |
| R-29 | S11-D12 | Inferior Frontal | 6.04 | 1.09 | 5.53 | <0.001 | <0.001 |
| R-30 | S12-D9 | Inferior Frontal | 6.31 | 1.15 | 5.51 | <0.001 | <0.001 |
| R-27 | S11-D9 | Inferior Frontal | 3.51 | 0.76 | 4.60 | <0.001 | <0.001 |
| R-39 | S15-D13 | Superior Temporal | 5.55 | 1.23 | 4.53 | <0.001 | <0.001 |
| R-34 | S13-D10 | Middle Temporal | 7.51 | 1.72 | 4.36 | <0.001 | <0.001 |
| R-37 | S14-D13 | Middle Temporal | 3.41 | 0.87 | 3.92 | <0.001 | <0.001 |
| R-42 | S16-D14 | Middle Temporal | 3.50 | 0.94 | 3.71 | <0.001 | <0.001 |
|  | | | | | | | |
| *Affixes condition* | | | | | | | |
| L-10 | S5-D2 | Inferior Frontal | 4.16 | 0.90 | 4.64 | <0.001 | <0.001 |
| L-7 | S3-D5 | Inferior Frontal | 4.56 | 1.02 | 4.47 | <0.001 | <0.001 |
| R-42 | S16-D14 | Middle Temporal | 4.17 | 0.93 | 4.50 | <0.001 | <0.001 |

**Table S9**

*English Monolinguals’ Brain Behavior Association with English Vocabulary*

| **Hem.-Ch#** | **Source-Detector** | **Region** | ***β*** | ***se*** | ***t*-stat** | ***p*** | ***q*** |
| --- | --- | --- | --- | --- | --- | --- | --- |
| *Free roots/compounds condition* | | | | | | | |
| L-16 | S7-D5 | Middle Temporal | 5.99 | 0.98 | 6.13 | <0.001 | <0.001 |
| R-29 | S11-D12 | Inferior Frontal | 6.46 | 1.10 | 5.88 | <0.001 | <0.001 |
| R-31 | S12-D11 | Precentral | 4.70 | 0.99 | 4.73 | <0.001 | <0.001 |
| R-36 | S14-D12 | Middle Temporal | 4.82 | 1.05 | 4.59 | <0.001 | <0.001 |
| R-32 | S12-D12 | Inferior Frontal | 2.97 | 0.71 | 4.21 | <0.001 | <0.001 |
|  |  |  |  |  |  |  |  |
| *Affixes condition* | | | | | | | |
| L-11 | S5-D4 | Precentral | 3.92 | 0.91 | 4.33 | <0.001 | <0.001 |
| L-7 | S3-D5 | Inferior Frontal | -4.72 | 1.15 | -4.11 | <0.001 | <0.001 |
| L-18 | S7-D7 | Middle Temporal | -4.26 | 1.05 | -4.07 | <0.001 | <0.001 |
| R-31 | S12-D11 | Precentral | 4.17 | 0.97 | 4.28 | <0.001 | <0.001 |
| R-25 | S10-D9 | Middle Frontal | -3.34 | 0.86 | -3.86 | <0.001 | <0.001 |
| R-40 | S15-D14 | Middle Temporal | -3.70 | 0.95 | -3.88 | <0.001 | <0.001 |

**Table S10**

*Chinese-English Bilinguals’ Brain Behavior Association with Chinese Vocabulary*

| **Hem.-Ch#** | **Source-Detector** | **Region** | ***β*** | ***se*** | ***t*-stat** | ***p*** | ***q*** |
| --- | --- | --- | --- | --- | --- | --- | --- |
| *Free roots/compounds condition* | | | | | | | |
| L-7 | S3-D5 | Inferior Frontal | -4.20 | 0.66 | -6.34 | <0.001 | <0.001 |
| L-5 | S3-D2 | Inferior Frontal | -2.86 | 0.46 | -6.17 | <0.001 | <0.001 |
| L-6 | S3-D3 | Inferior Frontal | -3.19 | 0.65 | -4.89 | <0.001 | <0.001 |
| L-9 | S4-D4 | Middle Frontal | -2.23 | 0.51 | -4.40 | <0.001 | <0.001 |
| L-14 | S6-D4 | Precental | 2.86 | 0.71 | 4.02 | <0.001 | <0.001 |
| L-3 | S2-D2 | Middle Frontal | -1.87 | 0.47 | -3.94 | <0.001 | <0.001 |
| L-15 | S6-D6 | Postcentral | 2.11 | 0.53 | 3.97 | <0.001 | <0.001 |
| R-31 | S12-D11 | Precentral | 3.16 | 0.55 | 5.71 | <0.001 | <0.001 |
| R-32 | S12-D12 | Inferior Frontal | 1.90 | 0.44 | 4.29 | <0.001 | <0.001 |
| R-28 | S10-D11 | Middle Frontal | 2.62 | 0.63 | 4.17 | <0.001 | <0.001 |
|  | | | | | | | |
| *Affixes condition* | | | | | | | |
| L-10 | S5-D2 | Inferior Frontal | 2.72 | 0.51 | 5.30 | <0.001 | <0.001 |

**Table S11**

*Chinese-English Bilinguals’ Brain Behavior Association with Chinese Morphological Awareness*

| **Hem.-Ch#** | **Source-Detector** | **Region** | ***β*** | | ***se*** | ***t*-stat** | ***p*** | ***q*** |
| --- | --- | --- | --- | --- | --- | --- | --- | --- |
| *Free roots/compounds condition* | | | | | | | | |
| L-7 | S3-D5 | Inferior Frontal | | -43.38 | 7.56 | -5.74 | <0.001 | <0.001 |
| L-5 | S3-D2 | Inferior Frontal | | -25.06 | 5.17 | -4.85 | <0.001 | <0.001 |
| L-14 | S6-D4 | Precental | | 41.04 | 8.74 | 4.70 | <0.001 | <0.001 |
| R-31 | S12-D11 | Precentral | | 59.90 | 6.68 | 8.96 | <0.001 | <0.001 |
| R-28 | S11-D10 | Inferior Frontal | | 48.39 | 7.33 | 6.60 | <0.001 | <0.001 |
| R-27 | S11-D9 | Inferior Frontal | | 27.71 | 5.08 | 5.46 | <0.001 | <0.001 |
| R-34 | S13-D10 | Middle Temporal | | 52.87 | 12.41 | 4.26 | <0.001 | <0.001 |
|  | | | | | | | | |
| *Affixes condition* | | | | | | | | |
| L-7 | S3-D5 | Inferior Frontal | | 62.21 | 7.12 | 8.73 | <0.001 | <0.001 |
| L-11 | S5-D4 | Precentral | | 28.95 | 5.60 | 5.17 | <0.001 | <0.001 |
| L-3 | S2-D2 | Middle Frontal | | 23.48 | 4.97 | 4.73 | <0.001 | <0.001 |
| L-6 | S3-D3 | Inferior Frontal | | 31.99 | 7.08 | 4.52 | <0.001 | <0.001 |
| L-10 | S5-D2 | Inferior Frontal | | 25.15 | 5.89 | 4.27 | <0.001 | <0.001 |

**Figure S1**

Subject-level Beta Value Distribution for the Significant Channels by Group by Condition

**
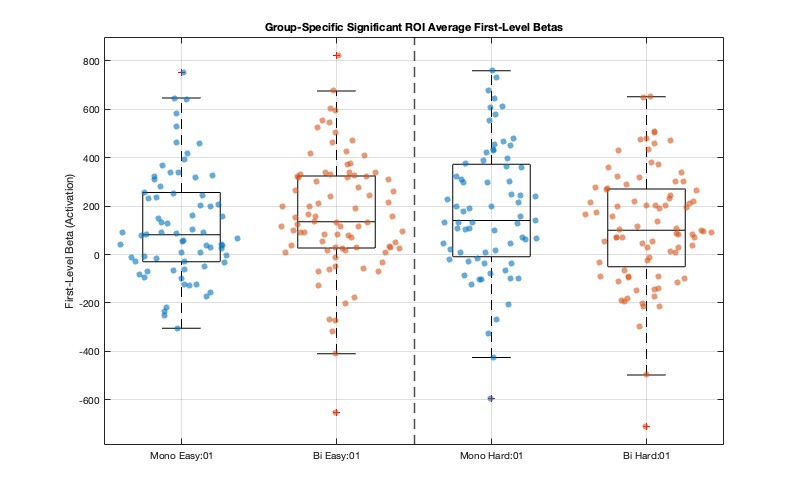
**

**Figure S2**

Participants’ Right Hemisphere Activation during Free Roots/Compounds and Affixes (task > rest, all FDR adjusted *q* < .001)

**Figure S3**

Control Condition Activation (all FDR adjusted *q* < .001)

**
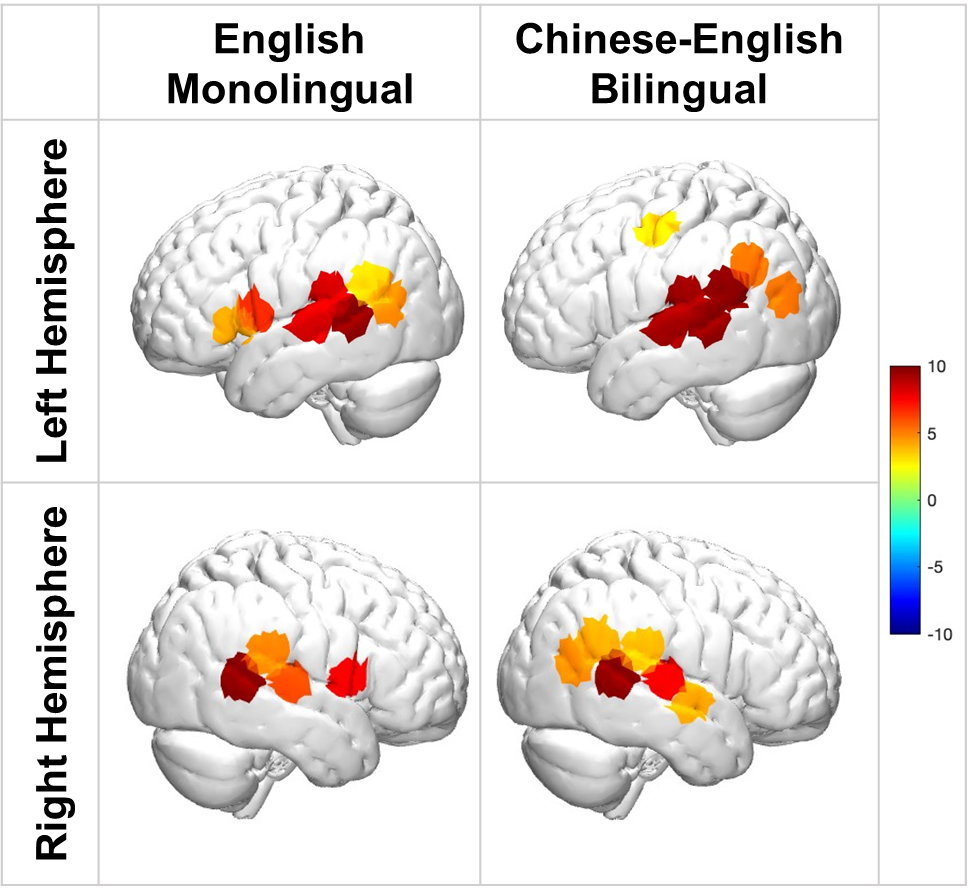
**

**Figure S4**

Right Hemisphere Brain-behaviour Associations Between English Morphological Processing and English Vocabulary by group and condition (all FDR adjusted *q* < .001)

**Figure S5**

Right Hemisphere Brain-behaviour Associations Between English Morphological Processing and Chinese Morphological Awareness and Chinese Vocabulary by task condition (all FDR adjusted *q* < .001)
